# Competition between Photoinduced Electron Transfer and Resonance Energy Transfer in an Example of Substituted Cytochrome c–Quantum Dot Systems

**DOI:** 10.1021/acs.jpcb.1c00325

**Published:** 2021-03-24

**Authors:** Jakub Sławski, Rafał Białek, Gotard Burdziński, Krzysztof Gibasiewicz, Remigiusz Worch, Joanna Grzyb

**Affiliations:** † Department of Biophysics, Faculty of Biotechnology, University of Wrocław, ul. F. Joliot-Curie 14a, 50-383 Wrocław, Poland; ‡ Faculty of Physics, Adam Mickiewicz University in Poznań, ul. Uniwersytetu Poznańskiego 2, 61-614 Poznań, Poland; § Institute of Physics, Polish Academy of Sciences, Al. Lotników 32/46, 02-668 Warsaw, Poland

## Abstract

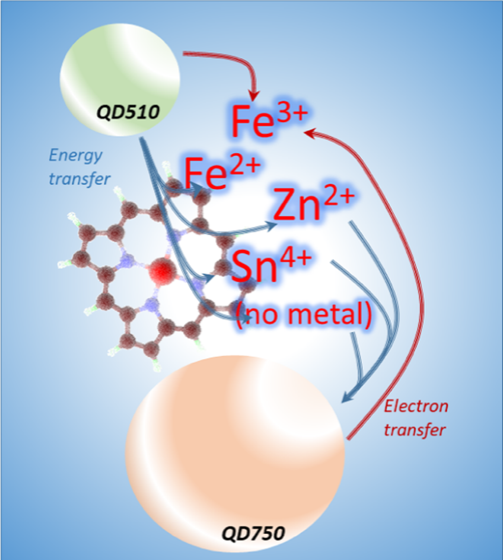

Colloidal quantum dots (QDs) are nanoparticles that are able to photoreduce redox proteins by electron transfer (ET). QDs are also able to transfer energy by resonance energy transfer (RET). Here, we address the question of the competition between these two routes of QDs’ excitation quenching, using cadmium telluride QDs and cytochrome c (CytC) or its metal-substituted derivatives. We used both oxidized and reduced versions of native CytC, as well as fluorescent, nonreducible Zn(II)CytC, Sn(II)CytC, and metal-free porphyrin CytC. We found that all of the CytC versions quench QD fluorescence, although the interaction may be described differently in terms of static and dynamic quenching. QDs may be quenchers of fluorescent CytC derivatives, with significant differences in effectiveness depending on QD size. SnCytC and porphyrin CytC increased the rate of Fe(III)CytC photoreduction, and Fe(II)CytC slightly decreased the rate and ZnCytC presence significantly decreased the rate and final level of reduced FeCytC. These might be partially explained by the tendency to form a stable complex between protein and QDs, which promoted RET and collisional quenching. Our findings show that there is a net preference for photoinduced ET over other ways of energy transfer, at least partially, due to a lack of donors, regenerating a hole at QDs and leading to irreversibility of ET events. There may also be a common part of pathways leading to photoinduced ET and RET. The nature of synergistic action observed in some cases allows the hypothesis that RET may be an additional way to power up the ET.

## Introduction

In nature, electron transfer (ET) (including photoinduced ET) plays an important role in cellular processes. When some artificial particles, such as colloidal quantum dots (QDs), are introduced in natural systems, they may compete with natural electron donors and acceptors. This situation becomes more complicated when the fluorescence properties of QDs are included; they introduce the possibility of resonance energy transfer (RET) from/to QDs. It also means that one particle may participate in both ET and RET. Here, we intend to define the rules governing the energy routes, using cytochrome c and its fluorescent derivatives as donors or acceptors for QDs in ET and RET.

QDs are semiconductor nanoparticles, which have attracted the attention of people working in the medical and life sciences fields. QDs are used in imaging as well as in various biosensing assays. The main reason for this is their broad absorption and narrow emission spectra. Other important features are the high fluorescence quantum yield and high resistance to photobleaching. Due to the broad absorption spectra, QDs are relatively easy to use in multiplexes, when one laser line may excite QDs of several colors.^1,2^ Biosensors with QDs are mostly based on Förster resonance energy transfer (FRET) between them and fluorescent dyes.^3−5^ In some such assays, however, ET between redox-active molecules and QD is responsible for analyte detection. There, the ET changes the intensity of QDs’ fluorescence. Interestingly, the same type of QDs, e.g., cadmium telluride (CdTe) nanoparticles, may be used in both types of sensors, based on excitation energy and ET.^6−8^ Algar and co-workers^9^ have shown, on the example of CdSe/ZnS QDs, labeled with ruttenium(II) phenantroline and fluorescent dyes, competition between charge transfer and FRET.

QDs may be a source of photoinduced ETs for the reduction of various molecules, from small chemicals, like dihydrophenylalanine,^10^ to redox-active proteins, such as cytochrome c (CytC)^11,12^ and ferredoxin.^12^ Photoinduced ET rate and photoreduction efficiency were shown to be related to the QD diameter and to the energy of QDs’ conduction band (CB). We also proved that the photoinduced electrons come from both conduction bands (CBs) and nanoparticle defects.^13^ For these reasons, QDs may potentially be applied in the construction of light-driven nanodevices for controlling cellular homeostasis. In a recent review, we broadly described the involvement of nanoparticles, including QDs, in both ET and RET.^14^

Cytochromes, which basically means cellular pigments,^15^ are reddish proteins with heme as their prosthetic group. CytC, which is the subject of this paper, is a soluble protein involved in mitochondrial respiration.^16^ It transfers electrons between two membrane protein complexes: cytochrome bc1 and terminal oxidase. CytC is also involved in several other cell processes, including apoptosis regulation, scavenging of reactive oxygen species (ROS) as well as ROS production.^17,18^ Catalytic activity of CytC toward plasmenylcholine and plasmenylethanolamine has been also shown.^19^ Very recently, CytC has been proven to be able to work in a redox cycle, involving gold nanoparticles and tannic acid (molecular oxidant).^20^ Most of these functions are related to the redox reaction of CytC. Electron shuttling is thus possible due to the presence of the heme moiety. When the electron is accepted, the central iron atom of heme is reduced from Fe^+3^ to Fe^+2^. This process is accompanied by a change in the CytC absorption spectrum—redshift—and an increase of the extinction coefficient of the Soret band, as well as intensification of absorption in the Q band.

In CytC, the heme moiety is bound via a covalent thioether bond to a Cys–X–X–Cys–His peptide motif. Additionally, the central Fe atom is coordinated by histidine. In bovine CytC, the sixth coordinate for Fe is provided by the methionine residue. In CytCs from other sources, it may be histidine, or there is no 6th ligand.^21^ The midpoint potential (*E*_m_, the redox potential of the electrode when the activities of the reductant and oxidant are equal; here, it means the equal concentration of reduced and oxidated protein) depends on the protein environment of the heme moiety and is one of the factors defining a particular cytochrome position in ET chains.

A prosthetic group of cytochromes, heme, due to the presence of iron, is nonfluorescent in either its free or its bound version. CytC is also an efficient fluorescence quencher. It is possible to obtain a substituted version of CytC,^22−26^ with iron replaced by other metal cations as listed in Table 1. They differ with respect to their redox activity and luminescent properties—zinc and tin CytCs are fluorescent, as well as a metal-free version of this protein (porphyrin CytC). Zinc-substituted CytC has been widely used in studies of ET between proteins^27^ as well as fluorescent derivatives of CytC in studies of localization and conformational changes upon lipid binding.^28,29^ Also, SnCytC was used in the construction of a photoelectric transducer.^30^ MnCytC and CoCytC are redox-active, with different redox properties from native FeCytC. These versions of the protein were used in the analysis of protein folding and probing of ET mechanisms.^31^ Substitution of the central heme atom was useful in studies of myoglobin and functional mimics of heme proteins.^32^

**Table 1:** Luminescence Properties and Redox Activity of CytC Protein and Its Derivatives

protein	luminescence	redox activity	references
FeCytC		redox-active (*E*_m_ = +286 mV)	(33)
porphCytC	emission from singlet (τ = 6.5 ns) state	redox-inert (no metal)	(22)
ZnCytC	emission from singlet (τ = 3.2 ns) and triplet (τ = 10 ms) states	redox-inert (Zn^II^)	(22, 23, 27)
SnCytC	emission from singlet (τ = 0.8 ns) and triplet states	virtually redox-inert, not reduced by dithionite (Sn^IV^)	(22)
CuCytC	fluorescence measurable in liquid nitrogen temperature	redox-inert (Cu^II^)	(23, 24)
CoCytC		redox-active (*E*_m_= −140 mV), the Co^III^ form is stable, Co^II^ can be prepared in the absence of oxygen	(26, 34)
MnCytC		redox-active (*E*_m_ = +60 mV), Mn^III^ is the main form, Mn^II^ is stable only in the excess of dithionate	(25, 26)

Here, we used CytC and its derivatives as model proteins to investigate the emerging question of energy transfer between them and QDs occurring via two coexisting routes, namely, photoinduced ET and (F)RET. It is important to understand such a competition for different donors/acceptors, which may coexist in cells or in any experimental assay with a complex environment. Both processes manifest as the quenching of donor fluorescence; however, their mechanisms are different.^35^ The aforementioned work of Algar et al.^9^ showed competition between ET and FRET using QDs labeled with small molecules. In our approach, fluorophores and redox-active molecules are cofactors of the same protein template. The only difference is that the central metal atom is in a heme moiety. We also allowed the free exchange of proteins at the QD surface, not introducing covalent binding CytCs. Therefore, the obtained system may be interpreted as closer to an in vivo situation, in which both ET and FRET partners are mostly proteins. The understanding of the competition between these two pathways is also crucial in terms of the performance of QDs in possible assays and applications in the presence of electron and energy acceptors such as CytC.

## Experimental Section

### Quantum Dots and Proteins

Hydrophilic CdTe QDs, coated with 3-mercaptopropionic acid with the fluorescence emission maximum at 510 nm (QD510), 550 nm (QD550), 630 nm (QD630), and 750 nm (QD750), were purchased from PlasmaChem. The diameters and molar extinction coefficients of QDs were calculated according to Yu et al.^36^ (see Table S1). Stock solutions of QDs were prepared in Milli-Q (MQ) water and stored at 4 °C in the darkness.

Bovine heart CytC was obtained from Sigma-Aldrich and was considered to be a fully oxidized form, with ε_410_ = 106.1 mM^–1^ cm^–1^.^13^ Fe(II)CytC was prepared by reduction with solid sodium dithionate followed by a buffer exchange, using a HiTrap desalting 5 mL column (GE Healthcare), to the deoxygenated (bubbled with nitrogen for at least 30 min) 25 mM *N*-(2-hydroxyethyl)piperazine-*N*′-ethanesulfonic acid (HEPES) at pH 7.5 and closed under nitrogen in the gas-tight vial. Preparation of porphyrin CytC (porphCytC) by iron removal and metal substitution to obtain ZnCytC and SnCytC were performed, as described by Vanderkooi et al.,^22^ from which molar extinction coefficients of CytC derivatives (see Table S1) were taken. Proteins were used as 20–30 μM stock solutions in 25 mM HEPES at pH 7.5. For absorption and emission spectra of proteins and QDs, see Figure S1. Spectral overlap values for QD–CytC pairs, and redox properties of QDs and CytCs (if available), are provided in Table S1.

Bovine serum albumin (BSA) was obtained from Sigma-Aldrich and was used as a 50–100 μM stock solution in 25 mM HEPES at pH 7.5 (ε_280_ = 43.8 mM^–1^ cm^–1^, according to the supplier directions). Zinc protoporphyrin IX (ZnPP) was purchased from Sigma-Aldrich and was stored as concentrated stock in dimethyl sulfoxide (DMSO) and was diluted (∼500×) with 25 mM HEPES at pH 7.5 before use (ε_407_ = 34.8 mM^–1^ cm^–1^).^37^ Alexa Fluor 488 dye was purchased from ThermoFisher.

### Illumination Experiments

The concentrations of the compounds were 0.1 μM QD510/QD550 and 1 μM of each CytC form present. After preparation, 1 mL of the samples was split into two parts: the first part was incubated in the darkness for 30 min as a control. The second part was illuminated by a 9 W low-pressure mercury-vapor lamp through a 300–400 nm band-pass filter. During illumination, absorption spectra of samples were measured every 5 min for 30 min using a DU 800 spectrophotometer (Beckman Coulter). After 30 min of incubation, the absorption of the darkness-incubated sample was also measured. The increase of 548 nm absorption during illumination was considered as an indicator of the Fe(III)CytC reduction process. Peaks at 548 nm were small and not easily distinguishable from measured spectra. In addition, direct absorbance read-out was difficult due to an inconstant background (QD absorption was changing during illumination); hence, subtraction of the baseline (outlying parts of the spectra) was necessary to extract 548 nm peaks. The absorbance after the addition of a saturating amount of sodium dithionate was considered to represent a maximal reduction level of CytC. The obtained 548 nm absorbance values of illuminated samples were used to plot the time courses of the reduction process. The 548 nm absorbance of darkness-incubated controls represented a reduction level without illumination and was used to create a linear background, which was subtracted from the reduction-in-time plots for illuminated samples (Figure S2).

### Titration Experiments

Titrations were performed in a quartz cuvette (5 mm × 10 mm) at 20 °C (unless otherwise stated) and kept in the spectrofluorometer holder. Small (2–3 μL) volumes of titrant were added to 500 μL of the sample solution buffered by 25 mM HEPES at pH 7.5. Concentrations of titrated QDs were 0.5 μM QD510 or 0.01 μM QD750, and proteins were added in 0.05–0.1 μM steps to the final concentration of 0.5 μM (protein/QD molar ratio equal to 1:1 and 50:1, respectively); in the cases of Fe(III)CytC and Fe(II)CytC, the final protein concentration was 0.25 μM due to the much higher quenching efficiency of these CytCs. When QDs were titrated by mixtures of different CytC forms, the final concentration of each protein was 0.5 μM (the total protein concentration was doubled). In the case of CytC derivatives titrated by QDs, the CytC concentration in the cuvette was 1 μM, and QDs were added in 0.1–0.2 μM (QD510) or 0.001–0.002 μM (QD750) steps to the final concentration of 1 μM QD510 or 0.01 μM QD750. All of the QDs and protein concentrations were adjusted to obtain the appropriate fluorescence intensity in terms of fluorimeter detector sensitivity, convenient measurement of spectral changes, and sufficient resolution of individual compounds’ spectra.

### Fluorescence Measurements

Steady-state and time-resolved fluorescence was measured using an FS5 spectrofluorometer (Edinburgh Instruments, U.K.) equipped with a thermostatic sample holder (water bath regulated), a TCSPC detector, a xenon lamp, and a 404 nm pulsed diode laser. The lifetime measurement time span was adapted to the average lifetime value of the respective compound and was equal to 50 ns (ZnCytC and SnCytC), 100 ns (porphCytC and QD510), or 500 ns (QD750), which corresponded to 20, 10, or 2 MHz repetition frequency. The laser operated at a typical peak power per pulse of 110 mW, and the emission bandwidth was set at 5 nm. The excitation wavelength was 404 nm for all of the spectral and lifetime measurements. Spectra were collected in the 420–800 nm emission range. The selected emission wavelengths of lifetime measurements were 510 nm for QD510, 750 nm for QD750, 615 nm for porphCytC, 580 nm for ZnCytC, and 574 nm for SnCytC. Details of data fitting are provided in Supporting Information.

### Gel Filtration

For the gel filtrations, a Superdex 200 5/150 GL (GE Healthcare) column was used. The column was connected to the ÄKTA Purifier chromatography system (GE Healthcare) and equilibrated with a buffer: 25 mM phosphate buffer at pH 7.4, or 100 mM phosphate at pH 7.4, or 25 mM HEPES, 50 mM NaCl at pH 7.4. The samples were loaded using a 100 μL loop, and the elution profiles were recorded by an internal spectrophotometric detector. The absorbance for two wavelengths was measured: 280 and 350 nm (in the case of BSA samples) or in the range of 397–412 nm, depending on the Soret absorption maximum of loaded CytC species. For determination of hydrodynamic radii, an LMW gel filtration calibration kit (GE Healthcare) was used.

### Fluorescence Correlation Spectroscopy (FCS) Measurements

Fluorescence correlation spectroscopy (FCS) measurements were performed on a Zeiss 780 ConfoCor 3 microscope with a C-Apochromat 40× water immersion objective with a numerical aperture of 1.2. For excitation of fluorescent CytC derivatives and QDs, a 405 nm laser diode and a 488 nm line of argon laser were used, respectively. Emitted light was passed through a 495–555 nm band-pass filter (for QD510) or a 505 nm long-pass filter (for QD550, QD750, and CytC). In the case of cross-correlation measurements of QD510 and CytC mixtures, emission was split by an NFT565 secondary dichroic filter and passed through 495–555 nm BP (QD510) and 580 LP (CytC). An avalanche photodiode of a Zeiss 780 ConfoCor 3 unit was used as the detector. The measurements were performed on 20 μL droplets placed on a #1.5 glass cover slide of 170 ± 5 μm thickness (Roth, High Precision) at room temperature (23 °C). Details of data analysis are provided in Supporting Information.

## Results and Discussion

### Competition between Electron-Transfer and Resonance-Energy-Transfer Pathways: Theoretical Analysis

**Figure 1:**
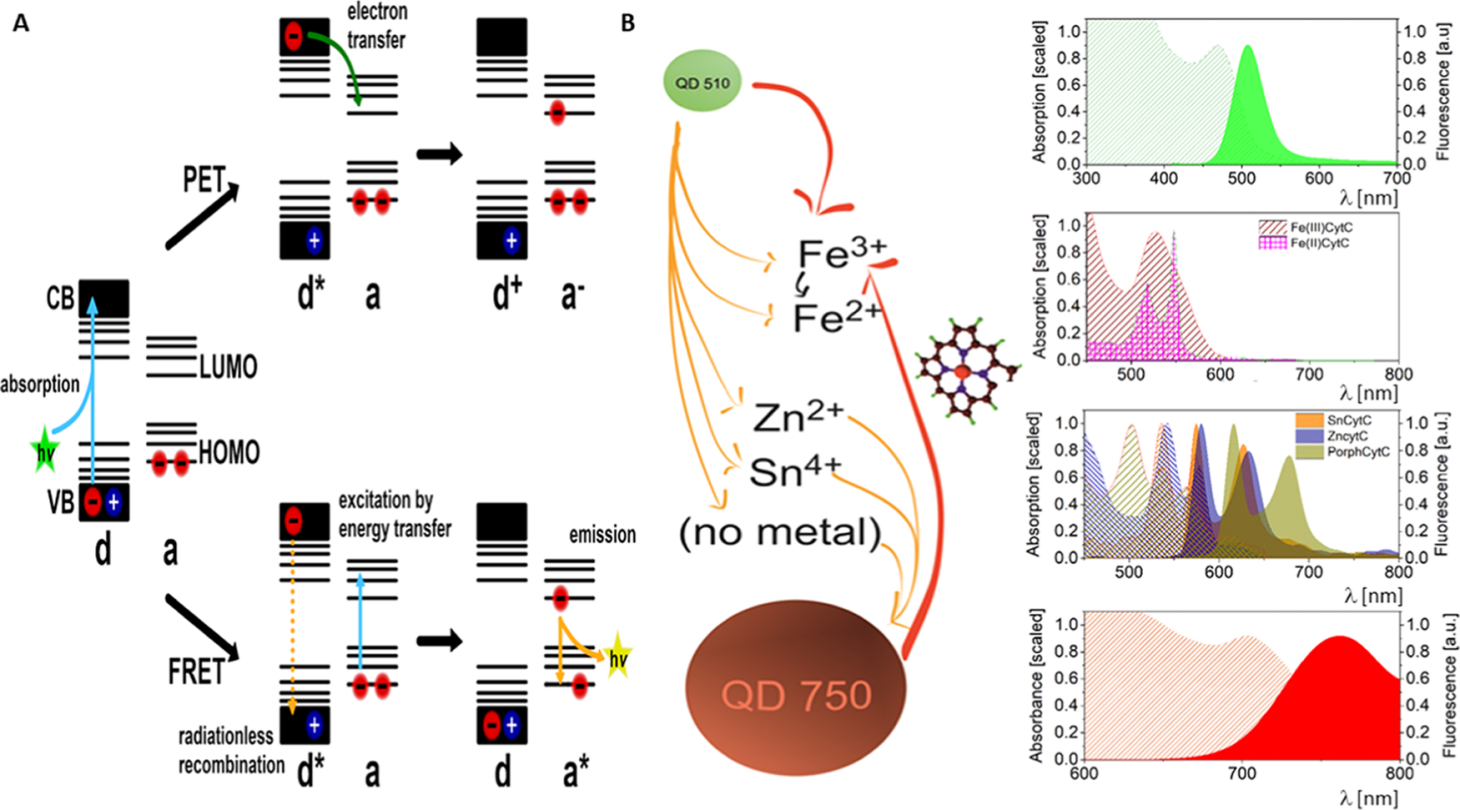
Schematic representation of (A) photoinduced electron transfer (PET) and resonance energy transfer (FRET) events with a QD donor (d) of energy or electrons and a molecular acceptor (a), and (B) possible means of PET (red arrows) and FRET (orange lines) events with QD510, QD750, and CytC versions—oxidized Fe(III), reduced Fe(II), metal-free porphyrin CytC, and a Zn^2+^- or Sn^4+^-substituted version. The terms conduction band (CB) and valence band (VB) were adopted for QDs as entities transient between solid-state materials and molecules of discrete energy levels (HOMO, highest occupied molecular orbital; LUMO, lowest unoccupied molecular orbital). The black arrow represents Fe(III)–Fe(II) transition after the ET event. Arrows point from the donor to the acceptor. Sphere sizes for QD representation correspond to differences in the QD diameter. The cytochromes are represented by metal only. The right side of the figure shows the absorption (hatched area) and emission (if possible, solid area) spectra of QDs and CytCs, which are aligned by wavelength.

As pointed out in the Introduction, nanoparticles may be involved in both photoinduced ET and RET, in particular via the Förster mechanism. The Dexter mechanism of RET is also possible^38,39^ and, in our experimental system, may be indistinguishable from FRET. The same type of nanoparticles, here CdTe QDs, may participate in both mechanisms, depending on the nature of the donor or acceptor. In most of the applications, a situation may easily occur in which both types of donors/acceptors are present simultaneously. Such competition is apparent for QDs in cell-like systems (e.g., in chloroplasts, with light-harvesting complexes, ferredoxin, and cytochrome proteins present), and it is also expected to occur in assays that contain more than one simple donor–acceptor pair. There, the question arises of whether one type of energy transfer is preferred over the other, and if so, what the rules are governing such a situation. To find the answers, we constructed assays composed of CdTe QDs and CytC or that of its metal-substituted derivatives. Fe(III)CytC is able to be an acceptor of an electron, while Zn/Sn/metal-free CytCs are nonreducible and fluorescent. Using CytC derivatives, we achieved an experimental setup in which the only variable was a mechanism of energy transfer (RET, via the Förster or Dexter mechanism, or photoinduced ET) between the donor and acceptor. Therefore, Fe(III)CytC may participate in both ET and energy transfer (mostly fluorescence quenching), and Zn/Sn/metal-free porphCytC should participate in energy transfer only. The efficiency of FRET depends both on the spectral overlap of the donor and acceptor and on the physical distance between them. On the basis of absorption and emission spectra of CytCs and QDs and assuming a value of dipole orientation factor (commonly denoted κ^2^) equal to 2/3, we calculated Förster distances (*R*_0_ is the distance at which the energy-transfer efficiency is 50%) of the possible donor–acceptor pairs (Table S1). The values in the range 2.85–6.27 nm indicate that FRET between QDs and CytC forms used in our research is possible, taking into account the diameters of the QDs, both theoretical (Table S1) and obtained from our experiments. The presented experiments gave insights into the competition between ET and RET. In our system, we purposely decided not to introduce covalent binding between QDs and CytCs. This secured the opportunity for protein molecule exchange at the QD surface, which is necessary for energy-transfer competition. Consequently, the donor–acceptor distance and orientation of the Förster/Dexter equation cannot be effectively defined. Yet, let us start by analyzing the situation when QDs are energy donors. To begin with, it is important to understand that processes of further energy transfer via ET or other mechanisms originate from the same event—absorption of a photon by a QD and an increase in the energy level of its electron with exciton (a pair of an electron, e^–^, and a hole, h^+^) generation. First, there is a possibility of simple relaxation, due to a recombination between an electron and a hole, and a molecule returning to its ground state with photon emission. The second option involves ET to the lowest unoccupied molecular orbital (LUMO, or, for the QDs, CB) of an acceptor. If an electron from a donor is transferred, then RET cannot occur until the donor is somehow reduced (Figure 1). An electron, by leaving a donor, generates a hole (+), which excludes any other events until the oxidized donor molecule is reduced, meaning that a hole is recombined with an electron from an external donor. This mechanism is true for one-electron donors, which are the majority of available natural donors. For two- or more-electron donors, two or more electrons may be transferred to acceptors. The ability of QDs to become photoinduced electron donors is not certain for fluorophores in general. There are molecules, such as flavin dinucleotide (FAD), which are fluorescent and may also participate in redox reactions.^40^ FAD, however, needs to be reduced by an external donor (e.g., nicotinamide adenine dinucleotide phosphate [NADPH] in an enzymatic reaction), and illumination is not a significant factor in this reaction.^41^

For the situation in which a QD works as a donor for FRET, the transfer event is possible if an appropriate acceptor is present within the transfer range during the lifetime of excitation. The recovery of the donor to the ground state is combined with a virtual photon emission. After that, there is no need for external donors to recover a QD. If a FRET acceptor is not present, the QD recombines to the ground state with or without fluorescence emission, and both ET and RET may occur after the next absorption event. If a QD was simply a one-electron donor, upon a single illumination event, it could undergo the first or the second scenario. Under continuous illumination, the presence of electron acceptors would gradually reduce the chance of FRET occurrence. For multiple-electron-donor QDs, the situation is similar, only extended via multiple possible absorption events. The number of electrons that can be transferred from a single QD is an open question. Experimental quantification suggests that a single QD may donate more than one electron (compare further photoreduction results, showing that 0.1 μM QDs may photoreduce at least 0.5 μM Fe(III)CytC, which means at least five electrons are donated from one QD). Therefore, we can assume that after donation of an electron, a QD may still be excited and may transfer energy via a resonance mechanism or donate another electron to an available acceptor. When photoreduction is carried out under continuous illumination, as in our experimental conditions, the time of total depletion of donors should be dependent on the diffusion rates, influencing the exchange of a reduced for an oxidized acceptor on the QD surface. In summary, the presence of FRET acceptors should reduce the photoreduction rate (inactivating QDs temporarily as electron donors until relaxation occurs); however, the final level (cumulative count of reduced acceptors) should not be impaired. We tested these hypotheses by following experimental approaches. For QDs as energy acceptors, which in our experimental system are represented by CytCs–QD750 mixtures, the picture is much simpler. There is no chance for ET in such a system because of two high *E*_m_ of reducible Fe(III/II)CytC. The other versions of CytCs are simple fluorophores. Even if a QD acceptor simultaneously accumulates excitation from more than one donor, this should result in a fluorescence increase proportional to the number of donors until they are a suitable distance away from the QD surface. A summary of possible means of energy transfer in our system is presented in Figure 1.

### CytC Derivatives Have Different Impacts on Fe(III)CytC Photoreduction by QDs

As we have already shown,^12,13^ CytC may be photoreduced by an electron originating from QD CB or QD crystal net defects. Here, we used the same assay^12^ to monitor the competition of nonreducible CytC forms (Fe[II]CytC, ZnCytC, SnCytC, and porphCytC) with the reducible one (Fe[III]CytC) for access to QD-originated energy. Fe(III)CytC reduction can be easily monitored spectrophotometrically due to changes in the absorption spectrum, reflecting the heme redox state. Different CytC forms present in a solution and competing for access to a QD surface possibly interfere with the process of Fe(III)CytC reduction. Hence, this competition (or lack of it) can be detected by the influence of additional CytC derivatives on spectral changes in the Q band of the native CytC absorption spectrum during light-induced reduction.

**Figure 2:**
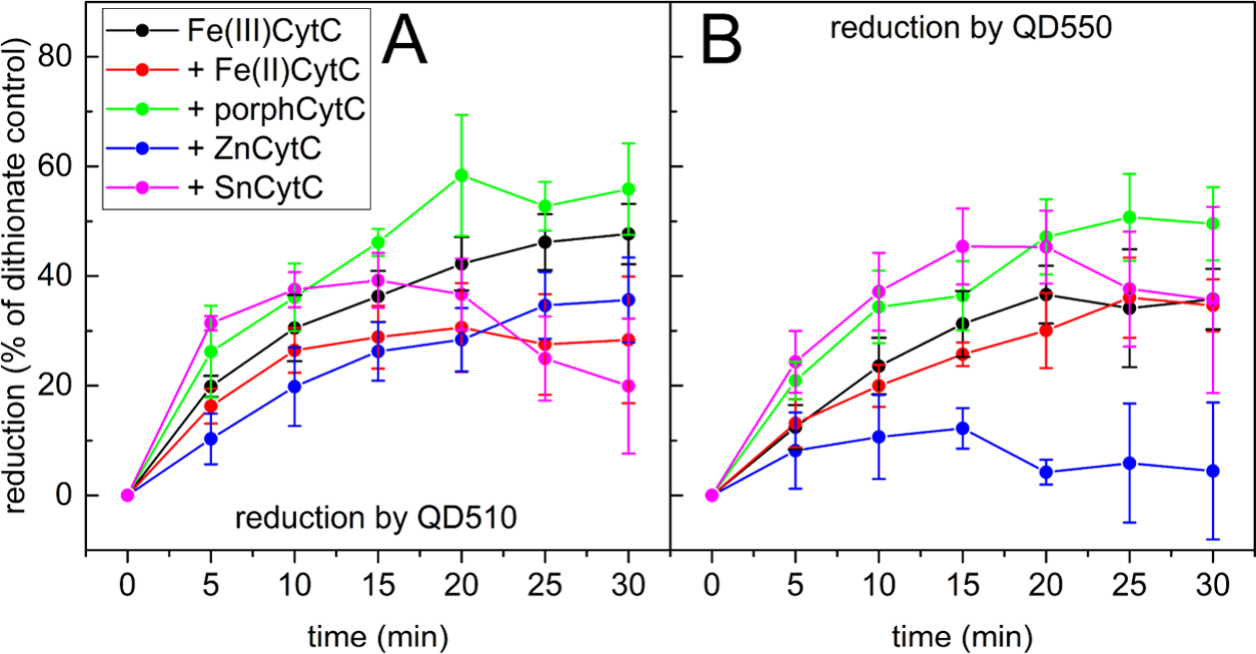
Photoreduction of Fe(III)CytC by illuminated QD510 (A) and QD550 (B) followed in time as an increase in 548 nm absorbance (dark reduction of CytCs was subtracted from the original data; compare Figure S2). The mixtures consisted of 0.1 μM QD510/QD550 and 1 μM of each protein in 25 mM HEPES at pH 7.5. Plots show the percent of reduction in reference to the sodium dithionate-treated Fe(III)CytC control. Error bars represent standard deviations of at least three independent repetitions.

Illumination experiments (Figure 2) indicated that UV-light-excited QDs reduced native Fe(III)CytC by 35–50% of the maximal reduction level (measured by the addition of a potent reducing agent, sodium dithionate). Larger QDs (QD550) are slightly weaker reducers than the smaller ones (QD510) (30–35% of the maximal reduction by dithionate). In a previous paper, QD750 was shown to reduce Fe(III)CytC,^12^ however, with a much lower efficiency than QD510 and QD550. Here again, the process of reduction by QD750 progressed at a significantly slower rate in comparison to QD510 or QD550 (Figure S3), and we decided not to include QD750 in further experiments due to an unclear analysis of the results.

The first important observation was that the presence of Fe(II)CytC or fluorescent, nonreducible derivatives of CytC had an effect on the reduction of Fe(III)CytC (Figure 2). This confirmed that there indeed may be competition between ET and RET from a QD to an acceptor. Surprisingly, porphCytC and SnCytC accelerated this process, and porphCytC increased the eventual cumulative level of the reduction by both QD510 and QD550. ZnCytC and, to a lesser extent, Fe(II)CytC had the opposite effects, which suggests competition for direct or indirect interaction with the QD surface. Some level of CytC reduction (10–50%) was achieved in the samples incubated with QDs in the darkness. It depended on an added CytC derivative and was especially high for SnCytC (up to 50%). The mechanism of change may be the same as the effect of SnCytC on a light-dependent reaction, as discussed in the following paragraph. This dark reduction may be attributed to ET from previously illuminated QDs with easily dispensable electrons, as observed previously.^12^ The background corresponding to this “dark” reduction was subtracted from the plots (Figure S2). In the presence of SnCytC, there was a noticeable drop after 15–20 min of illumination (Figure 2). This was the effect of the high level of “dark reduction” observed for SnCytC, not the actual reoxidation of CytC (compare with an example of the original data in Figure S2), and indicates the decline of the photoreduction rate in favor of light-independent reduction of CytC, which in the case of SnCytC is especially high.

An ANOVA and post hoc Tukey’s test showed significant difference (*p* < 0.05) only for the addition of ZnCytC with QD550 as a photoreductor (Table S2). However, there were clear trends suggesting differences in the time progression of photoreduction. To detect that, we also performed a post hoc T-test on pairs of Frechet distances of the curves (for a description, see Section 4 in the Supporting Information). This test indicated statistically significant differences (*p* < 0.05) for all cases, except for Fe(III)CytC–porphCytC in the presence of QD510 and Fe(III)CytC–Fe(II)CytC in the presence of QD550 (Table S3). The decrease in Fe(III)CytC reduction caused by ZnCytC may be explained by the FRET-type interaction of ZnCytC with QDs. It could be the result of a stable complex formation between ZnCytC and QDs and may be partly related to an increased possibility of dynamic quenching processes, including RET, which will be discussed in the following sections. The CytC with reduced iron, Fe(II)CytC, had a slight impact on the observed photoreduction. The small influence caused by Fe(II)CytC suggests that the presence of a reduced version of an acceptor did not significantly shift the Fe(III)/Fe(II)CytC redox equilibrium, and photoreduction was still a promoted direction. There were no indications that Fe(II)CytC might be an electron donor for QDs. An exchange of an electron between reduced and oxidized CytC versions in the solution was possible; however, it should not impair the measurement results and conclusions.

The increase in the photoreduction rate, observed here in the presence of porphCytC and SnCytC (Figure 2), cannot be simply explained. Fluorescent CytC derivatives are very unlikely to accept electrons. If such a situation were possible, it might, under some unlikely circumstances (a faster, more efficient reduction of a derivative than an exchange with Fe(III)CytC), result in the observed increase in the photoreduction rate. As might be predicted, FRET or electron acceptors (as well as broadly understood collisional quenchers) should be inhibitors of the photoreduction rate. We may then hypothesize that molecules of these CytC derivatives interact with QDs and prepare them for more efficient electron donation to the proper acceptor. It may occur in a way (e.g., by formation of charge-transfer complexes between QDs and CytC derivatives) that is similar to the behavior of dye-sensitized TiO_2_ nanoparticles.^42^ The second hypothesis assumes that porphCytC and SnCytC interact with the electron acceptor or its reduced version, Fe(III)CytC/Fe(II)CytC. Interaction with an electron acceptor may increase the photoreduction rate (e.g., by facilitating exchange at the QD surface). There is, however, no evidence that such effects take place.

We tried to characterize the kinetics of the photoreduction of Fe(III)CytC by QDs more precisely, performing a flash photolysis experiment (Figure S4). The approach takes advantage of absorption spectrum changes reflecting the redox state of FeCytC and allows assessment of the nature of linked physical processes. Unfortunately, a low level of CytC photoreduction under pulse illumination related to the bright emission of QDs (Figure S4A, fluorescence of QD630 with ∼100 ns time span) presented a substantial obstacle for the observation of spectral changes during the process of FeCytC reduction (Figure S4B, 416 nm wavelength chosen for the expected photoinduced absorption related to a 410–416 nm shift of the FeCytC Soret peak). ZnCytC triplet state absorption (Figure S4C) and photobleaching of the ground state (Figure S4D) were detected, confirming previous reports.^43,44^

Based on this part of the experiment, which examined the competition between parallel ET and RET processes, we can conclude that ET appears to dominate. This can be concluded simply because there was no drastic inhibition of the QD-dependent photoreduction of Fe(III)CytC. In the most significant case (ZnCytC and QD550), the inhibition was about 50%, and for other CytC derivatives, there was no change or increase in the rate. Because this explanation is based just on these data, it is worth distinguishing the irreversible process of QD electron donation to a CytC acceptor by an ET pathway from RET. The latter does not result in the transport of an excited electron and makes ET still possible in a subsequent excitation event, especially in conditions of constant illumination. Hence, even in the case of significant competition between ET and RET on a molecular level, the observable effects may support ET dominance due to its irreversibility.

### Fluorescence Quenching Analysis Reveals the Mechanism of Competition between Photoinduced ET and RET

#### Quenching of QD Fluorescence by CytC and Its Metal Derivatives

To better understand the interaction between QDs and CytC derivatives, we performed a titration of QDs with a protein solution. An example of an experimental set is shown in Figure S5. The titration showed that all of the CytC derivatives quenched the fluorescence of QDs in a concentration-dependent manner. The quenching was specific to CytCs as the composites of a polypeptide chain and its cofactor, since both BSA (a test for polypeptide chain interaction) and zinc protoporphyrin (a test for cofactor influence) have negligible effects on QD emission (Figure S6). For time-resolved measurements, a 404 nm excitation wavelength was chosen, which excites both QDs and fluorescent CytC derivatives. These effects were corrected by appropriate control measurements. The fitting of the obtained *F*_0_/*F* and τ_0_/τ Stern–Volmer curves to appropriate equations allowed for the calculation of dynamic (*K*_SV_) and static (*K*_a_) quenching constants. For calculation details, see the Supporting Information. The results revealed that the quenching mechanism and its efficiency depend on both the metal occupancy of CytC and the size of the QDs.

**Figure 3:**
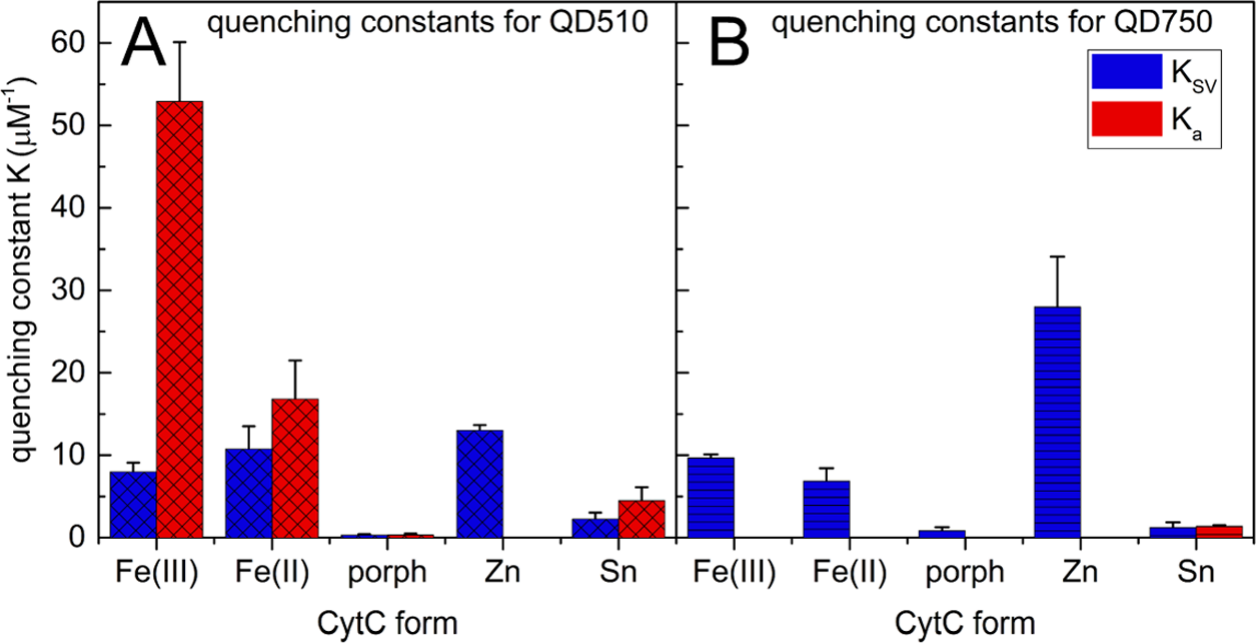
Comparison of dynamic (*K*_SV_) and static (*K*_a_) quenching constants calculated from the results of QD510 (A) and QD750 (B) titrations with cytochrome solutions. QD510 (0.5 μM) and QD750 (0.01 μM) in 25 mM HEPES at pH 7.5 were titrated with different CytC forms in 0–0.5 μM concentrations of protein. At every titration step, the QD fluorescence intensity and lifetime were measured (λ_ex_ 404 nm and λ_em_ 510 or 750 nm for QD510 and QD750, respectively). Error bars represent standard deviations of at least three independent repetitions.

Generally, a combined mechanism of QD fluorescence quenching by some CytC species dominates for the smaller QD510 (Figure 3A, both *K*_SV_ and *K*_a_ are in the measurable range), while the larger QD750 is quenched mainly dynamically (Figure 3B, only *K*_SV_ had a measurable value in most cases). The static component of QD510 quenching is the most significant factor in the presence of Fe(III)CytC (*K*_a_ = 53 μM^–1^). It also occurs for Fe(II)CytC (*K*_a_ = 17 μM^–1^) and, to a much lesser extent, for porphCytC and SnCytC derivatives (Figure 3). Temperature dependence of quenching confirmed these conclusions (see the relevant paragraph in the Supporting Information; also Figure S7). The number of theoretical binding sites (Figure S8) on the QD510 surface approximately equals two for both redox forms of native CytC and is less than one for porphCytC and SnCytC.

In contrast, QD750 is quenched purely dynamically by different species of CytC, with the exception of a small static component of the SnCytC derivative (*K*_a_ = 1 μM^–1^). The low theoretical number of binding sites for SnCytC (*n* = 0.6) is counterintuitive to the relatively large and multivalent surface of QD750 and may reflect the generally low quenching efficiency of SnCytC. The *K*_SV_ values calculated for QD750 are in the range of *K*_SV_’s for QD510, with the exception of ZnCytC (*K*_SV_ = 28 μM^–1^). This version of CytC is, however, the most effective dynamic quencher CytC for both QD types tested.

Since emission from both CB and defects of QDs contribute to the overall fluorescence of QDs, steady-state and time-resolved fluorescence measurement is an informative and straightforward means of their analysis.^45,46^ Previously, we demonstrated that Fe(III)CytC and ferredoxin are able to accept electrons from both the conduction band and surface traps.^13^ A more robust analysis of QD quenching by CytC derivatives may be accomplished by decomposition of QD emission decays to individual lifetime components (Figures S9 and S10). The multiexponential curve of QD lifetime contains at least three components, which were attributed to emission from the CB (the medium-length τ) and deep and shallow electron (or hole) traps (the shortest and the longest τ, respectively).^13^ During QD titration with Fe(III)CytC, not only did the relative contribution of decay components change but also the length of all components decreased (compare Figure S10), as observed previously.^13^ Since, by definition, static quenching does not affect the fluorescence lifetime, observed effects apply to the processes comprising the dynamic component of quenching.

In general, the average lifetime composed of single lifetime components of different lengths may decrease upon quenching through one or both of two different manners. First is the relative decrease of amplitude of longer components (and through that, the domination of shorter components) without a change of individual lifetime values (so for *I* = *I*_0_ + ∑ (*A*_i_ exp (−*t*/τ_i_))—see the explanation in the supplement—*A*_i_ values are altered and τ_i_ values are constant). This is demonstrated by shifts of percentile contribution of lifetime components into the average τ value (Figure S9). The second manner is the shortening of each lifetime component length without a relative change in its amplitudes (*A*_i_ values are constant, τ_i_ decreases; Figure S10). For QD quenching by CytCs, we observed both effects depending on the particular QD–CytC pair. This impact on the τ contribution ratio (contribution of the amplitudes of each component in the total fluorescence intensity) is the most strongly manifested by Fe(III)CytC, Fe(II)CytC, and ZnCytC. In contrast, QD750 dynamic quenching has a slightly greater influence on the τ-amplitude composition: the decrease in the average lifetime is mainly a result of the shortening of individual τ values, not a change in the τ contribution ratio. The exception here is ZnCytC, which shows a similar pattern of lifetime composition for both QD510 and QD750. Keeping in mind assumptions about individual τ components, we may postulate that, in contrast to QD core state electrons, surface-trapped electrons are efficiently relaxed by dynamic quenchers (Figure S9). The relative extent of this process on overall QD fluorescence may decrease with the decreasing surface/volume ratio of QDs, which is shown by a comparison of QD510 and QD750 quenching.

In summary, the high *K*_SV_ constant for ZnCytC suggests dynamic quenching as a possible cause of its competitive action toward simultaneous Fe(III) reduction, as shown in Figure 2. Lower values of *K*_SV_ for other CytC derivatives may explain their inefficiency in inhibiting ET, although this does not clarify the mechanism of the synergistic effect of porphCytC and SnCytC.

#### Effect of CytC Derivative Mixtures on QD Fluorescence

While the previous chapter describes a simple situation, with one transfer route (photoinduced ET or RET) clearly demonstrated, here we build the assay, allowing analysis of the interaction (competition or synergy) between those processes.

**Figure 4:**
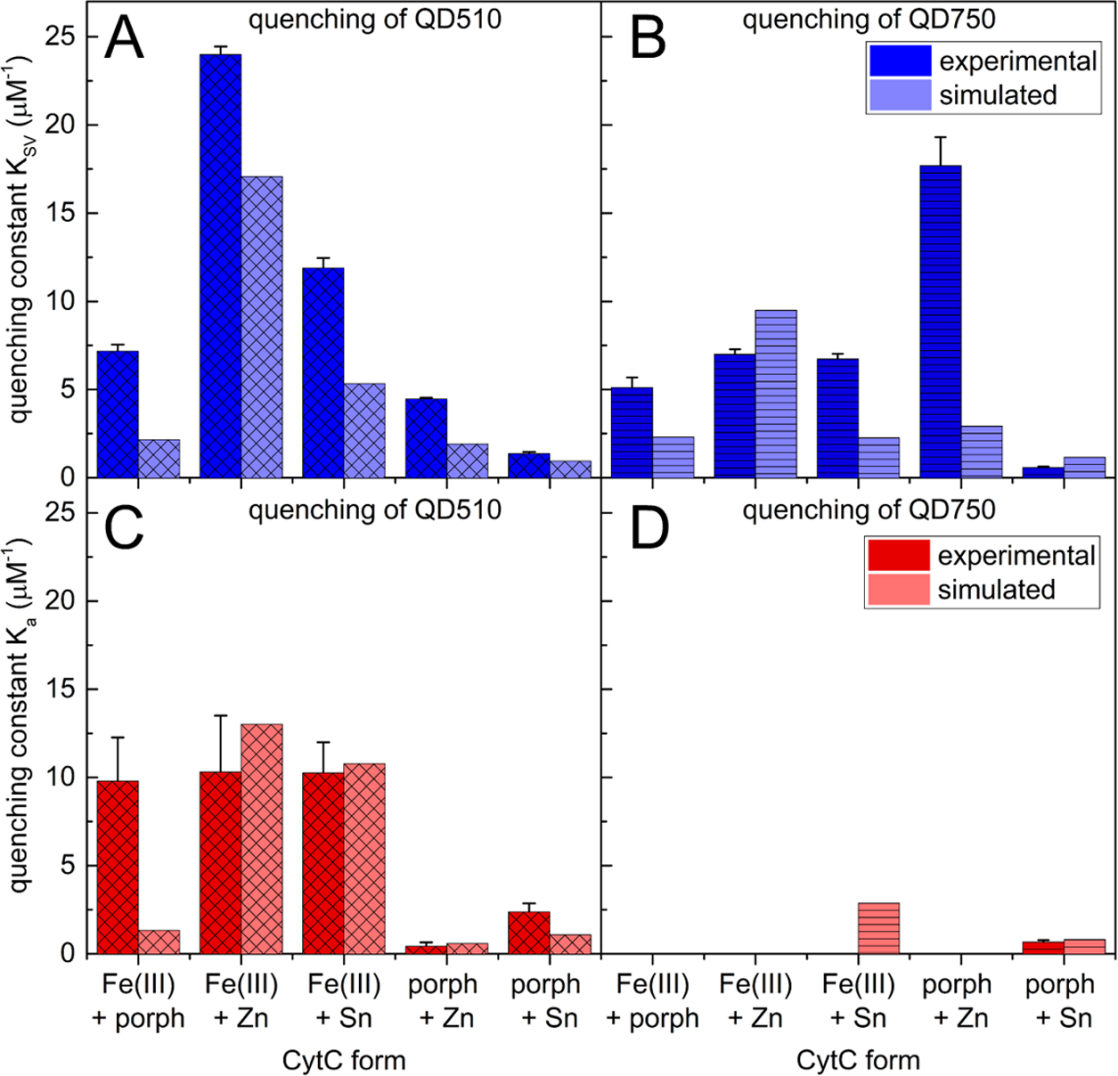
Comparison of the real and simulated quenching constants of QD510 (A, C) and QD750 (B, D) titrated with mixtures of different CytC forms. QD510 (0.5 μM) and QD750 (0.01 μM) in 25 mM HEPES at pH 7.5 were titrated with different CytC forms in concentrations of 0–0.5 μM (for mixes without Fe(III)CytC) or 0–0.25 μM (for mixes with Fe(III)CytC) of each protein in the pair. Experimental parameters were as described in Figure 3. During titration, mixed CytCs were added into the equimolar concentrations, and constant values were calculated for the concentration of the total CytC protein, regardless of the form. The simulated constants were obtained by fitting the averaged *F*_0_/*F* and *T*_av0_/*T*_av_ data for different single CytC species. Error bars of experimental data represent standard deviations of at least three independent repetitions.

In pursuit of this aim, titrations of QD510 and QD750 with different CytC mixtures were performed. Specifically, solutions of porphCytC and Fe(III)CytC mixed with other CytC species in a molar ratio of 1:1 were used. Quenching constants calculated after fitting the obtained Stern–Volmer curves were analyzed to determine the quenching efficiency of combined CytC proteins. For comparison with experimental data, we adopted a model of different CytC forms interacting independently with QDs, making the assumption that each CytC molecule has equal access to the QD surface (Figure S12). The net effect of mixed CytC forms on QD fluorescence was evaluated by comparing experimental and so-called simulated quenching constants (Figure 4). The notion of simulated quenching constants, representing no interaction between ET and RET, is explained in the Supporting Information (Figure S12 and the relevant paragraph).

Overall, the character of the simultaneous action of mixtures of different CytC forms varies depending on the QD size and CytC species. In the case of the dynamic component of the quenching of QD510, mixed CytC derivatives seemed to exhibit a synergistic effect on QD510 fluorescence (Figure 4A), as experimental constants exceeded the theoretical values. A possible explanation may be the competitive behavior of CytC forms with higher dynamic quenching efficiency, specifically Fe(III)CytC and ZnCytC. This would result in a crowding-out of the less efficient quenchers (porphCytC and SnCytC) and in their larger impact on QD fluorescence. This trend is similarly maintained for the dynamic quenching of QD750 (Figure 4B), except for the Fe(III)CytC–ZnCytC and porphCytC–SnCytC pairs.

The effects of different CytC pairs on QD fluorescence were independent, which was the most evident in the static mechanism of the quenching of QD510 (Figure 4C). Fe(III)CytC, the most potent static quencher of QD510, was presumably responsible for this effect. The apparent decrease in quenching efficiency in the presence of its derivatives appeared to result only from the depleted concentration of Fe(III)CytC in the mixtures of CytC proteins. The exception was the excessive competition of Fe(III)CytC toward porphCytC, with an increase of about 5 times the value of *K*_a_ in comparison with the expected static quenching constant. This was parallel to the observed increase in the photoreduction rate (Figure 2). Consequently, we propose that metal-lacking porphyrin may behave as a protectant for QDs. Protoporphyrin IX (iron-free heme) is known to be a pro-oxidant and a photosensitizer.^47^ It should be noted, however, that the laser power used in our experiment was greatly below that usually used in laser photolysis experiments that generate porphyrin radicals. There has also been a report showing iron-free porphyrin action against free radicals.^48^ Additionally, chemically similar pheophytins were shown to be efficient antioxidants.^49^ Thus, the action of porphCytC may be a result of it taking over possible radical states generated in the QD, e.g., by disproportionation reaction or by the reaction of porphCytC with residual oxygen present in the solution, protecting QDs from such a reaction.

In the assay containing ZnCytC and porphCytC with no ET possible, dynamic quenching of QD510 also increased (Figure 4A), suggesting that stabilization caused by porphCytC is indeed on the QD level and is important for the common part of QD-dependent energy transfer. For QD750-containing assays, where QDs are acceptors, the effect of porphCytC was even stronger (Figure 4B), which provides additional support for this stabilization theory. It was impossible to clearly analyze (discriminate amongst the components) the effect of QD-caused quenching for a mixture of CytC derivatives, so we cannot reinforce our conclusions based on this analysis. What is important here is that the static quenching of QDs is not restricted to ET. On the other hand, it seems to be much more significant for QD510 acting as a donor (for photoinduced ET or for other processes) than for QD750, with its acceptor-only function. This may correspond to photocorrosion, which has been observed in many previous cases.^50−52^ This is another argument for the preference of photoinduced ET as a de-excitation method, since photocorrosion of QDs contains the ET step in its mechanism.^51^

Similarly, as in the case of single-type CytC proteins, the mixtures do not exhibit static quenching of QD750 aside from a slight effect of the porphCytC–SnCytC pair (Figure 4D). The static component of QD750 quenching by SnCytC is not observable in the presence of Fe(III)CytC, which additionally confirms its competitive behavior. Despite the variable effects of CytC species acting in concert, the numbers of binding sites for mixtures were equal to the higher value of the two respective single CytC components, and they did not seem to be additive (Figure S8). An exception to this rule was the result for the Fe(III)CytC + porphCytC and Fe(III)CytC + ZnCytC mixtures, which both significantly exceeded the value of the two binding sites.

To sum up this part of experiments, mixed CytC derivatives exhibited a mostly synergistic effect on QD fluorescence. The hypothesis of a cross-link between RET and ET that can occur for QDs involved in those processes is supported by the differences between simulated and experimentally obtained quenching constants for the Fe(III)+porphCytC and Fe(III)+SnCytC pairs. Experimental values of *K*_SV_ are substantially lower than expected, especially for QD510 quenching (Figure 4A). This suggests that the presence of RET acceptors (porphCytC or SnCytC) may in some manner power up the ET rate between QDs and Fe(III)CytC, resulting in a much stronger ET-driven quenching than expected for Fe(III)CytC alone.

#### Quenching of Fluorescence of CytC Derivatives by QDs

**Figure 5:**
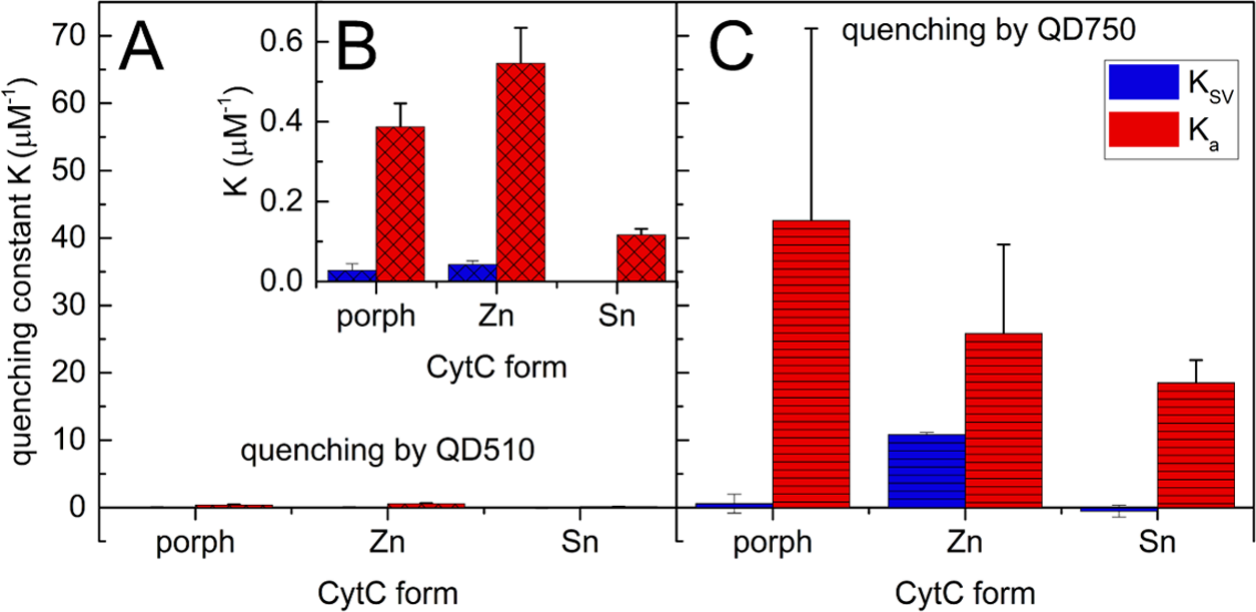
Quenching constant values for the quenching of fluorescent CytC derivatives by QD510 (A) and QD750 (C). For clarity, an inset showing the same results for QD510 as a quencher on a different scale is added (B). CytC solution (1 μM) was titrated with QD510 (0–1 μM) or QD750 (0–0.01 μM) and the fluorescence intensity and lifetime were recorded (λ_ex_ 404 nm, λ_em_ 615, 580, and 574 nm for porphyrin CytC, ZnCytC, and SnCytC, respectively). Error bars represent standard deviations of at least three independent repetitions.

Since the CytC derivatives used in this study, namely, porphCytC, ZnCytC, and SnCytC, have fluorescent properties, the question arose of whether QDs have any impact on the emission of these proteins. To examine that, the following experiments monitored by a spectrofluorometer were performed: CytC solutions were titrated with QD510 or QD750. In both situations, quenching of CytC emission was observed. The obtained quenching constants (Figure 5) showed that the quenching was primarily static with approximately one binding site on the CytC surface. QD750 is a much more potent quencher than QD510. The *K*_SV_ and *K*_a_ values for QD750 are 2 orders of magnitude higher than for QD510. Regardless of the size of the QD, they exhibit similar ratios of quenching constants (*K*_a_/*K*_SV_) for respective CytC derivatives.

For the titration of fluorescent CytC forms with QD510, being here a FRET donor, we actually expected an increase in the fluorescence intensity and lifetime of the fluorescent CytC derivatives, due to possible FRET. As this was not the case, we hypothesize that energy transfer is not fully FRET, and other dissipative processes are masking actual FRET. We also experienced signal contamination due to the coexcitation of a donor and an acceptor, as a result of limitations in available laser sets. Also, the less-than-optimal stoichiometry between donors and acceptors might have influenced measurements, as shown with fluorescein and rhodamine on the surface of silica nanoparticles.^53^ The same situation might be observed for free donors or acceptors in solution.^54^ A weak or no increase in the acceptor lifetime was observed for some FRET pairs, as for Alexa488–Alexa568 double-labeled apoflavodoxin.^55^ In that case, the fluorescence rise time was used as a criterion.

Fluorescence of ZnCytC and porphCytC was influenced similarly. Interaction with QDs may then induce a change in CytC molecules, e.g., conformational transition in the porphyrin cofactor neighborhood, which explains the domination of the static quenching mechanism.

An important question for further interpretation of our results is if we may assume that all of the static processes observed in quenching of CytC are the effect of ET and that dynamic processes are of FRET/resonance transfer only. This is definitely not the case, as for the quenching of fluorescent CytC derivatives (no ET possible^22^), we found domination of static quenching (Figure 5). Thus, static quenching might also be a result of, for example, conformational changes of the protein, which influences the fluorescence efficiency. Dynamic quenching was more likely to occur without an ET component, as the case of QD510 quenching by ZnCytC contained only a dynamic component. This was also confirmed by the observation of quenching characteristics under varied temperatures. However, we found that QD750 emissions were also quenched with dynamic characteristics. In ZnCytC–QD750, QDs are acceptors of energy, so their fluorescence should not be quenched but rather enhanced. This means that the component of quenching that we can assign as dynamic was not purely FRET but also contained other mechanisms of dynamic character, broadly defined as collisional quenching.

In summary, the mechanism of quenching of all CytCs’ fluorescence by QD510 or Qd750 was similar. Therefore, this is an indication that observed variations in Fe(III)CytC photoreduction do not directly depend on CytC de-excitation. The last factor that should be considered is the stability of the CytC–QD complex. A stable complex may facilitate energy or electron transfer. On the other hand, the QD surface is limited, and a too-stable complex with one protein may reduce the chance for interaction with another.

### Analysis of the Size and Stoichiometry of CytC–QD Complexes: Stable Complex Formation as a Possible Factor in Observed Processes

#### Gel Filtration of QD–CytC Mixtures

As previously shown by gel filtration and the dynamic light scattering method, native Fe(III)CytC does not form stable complexes with CdTe QDs in a phosphate buffer.^12^ It was not clear if CytC derivatives would share a similar behavior because metal substitution may change the overall charge distribution of the protein. Stable complex formation between CytC derivatives (especially ZnCytC) and QDs may influence the rate of exchange of acceptors (here, reduced Fe(II)CytC to oxidized ones) at the QD surface. To examine if the substitution of metal ions in the porphyrin cofactor may change the ability of CytC to bind to a QD surface, gel filtrations of CytCs–QD mixtures were performed. BSA was used as a positive control of protein–QD complex formation. The binding of BSA to QDs could be determined by the presence of a 350 nm absorption peak of the QD510–BSA complex at the elution volume of BSA (BSA alone does not absorb at this wavelength) or the peak at the void volume of the column corresponding presumably to the QD750–BSA multimeric complex or aggregate. This technique basically separates species by their hydrodynamic radii; the constituents with a higher radius elute faster, with a lower elution volume.

**Figure 6:**
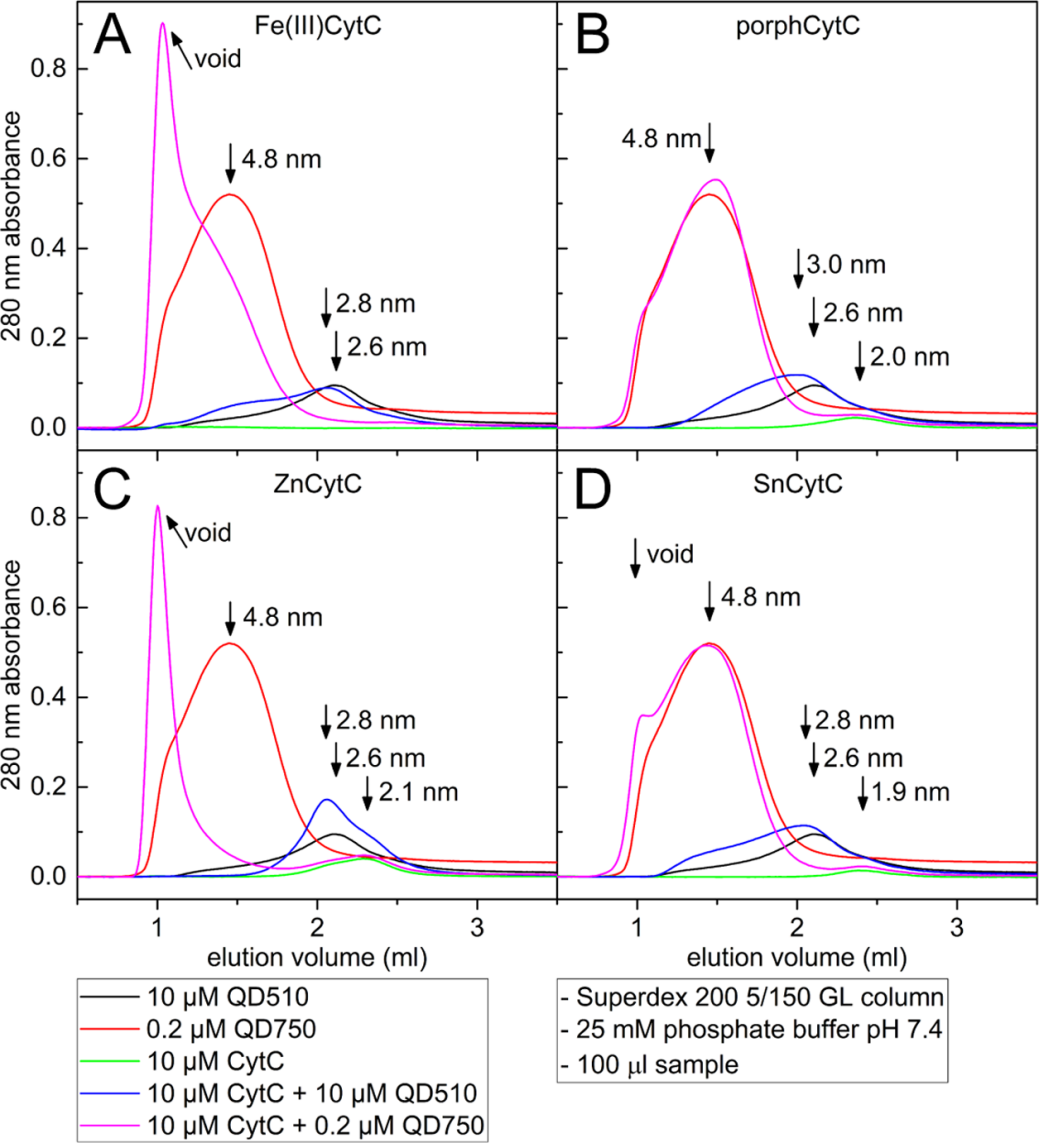
Chromatograms of gel filtrations recorded for Fe(III)CytC (A), porphCytC (B), ZnCytC (C), and SnCytC (D). Each set of samples included QD-only samples, CytC-only samples, and QD–CytC mixtures. The 100 μL samples were loaded on a Superdex 200 5/150 GL column in 25 mM phosphate buffer at pH 7.4. The concentration of proteins was 10 μM in 1:1 (QD510) or 50:1 (QD750) molar ratios to the QDs. The calculated hydrodynamic radii (or void volume of the column) are depicted.

Gel filtration of various QD–CytC mixtures (concentrations and ratios corresponding to final steps in QD–CytC titrations) in 25 mM phosphate buffer indicated significant differences (Figure 6). The hydrodynamic radii for pure QD510 and QD750 were determined as 2.6 and 4.8 nm, respectively. For QD750–CytC pairs, the shift of the elution peak position from the volume corresponding to a radius of 4.8 nm to the void volume of the column indicated the formation of large complexes/aggregates that were out of the separation range of the column. For subsequent CytC derivatives, the peak shift gradually increased: not observable for porphCytC (no change in the hydrodynamic radius of 4.8 nm), a slightly marked radius increase for SnCytC, and clear for Fe(III)CytC and ZnCytC. This order may illustrate the binding affinity between QD750 and subsequent CytC forms. The results for gel filtration of QD510 samples in the mixture with CytC proteins were not so indicative. All CytC derivatives, except for ZnCytC, induced broadening of the elution peak of QD510 and the formation of an asymmetric “tail” at larger elution volumes. This can be interpreted as QD510–CytC complex formation in the presence of some amount of QD510 remaining free. The chromatogram for the QD510–ZnCytC mix did not provide evidence of complex formation, as only overlapping QD510 and ZnCytC peaks could be observed. The same separations, monitored at the Soret maximum wavelength, confirmed our foregoing conclusions (not shown). The lack of an elution peak for pure Fe(III)CytC protein probably resulted from the stacking of the protein in the column bed in the low-ionic-strength buffer, which was 25 mM phosphate (which was also observed for other CytC derivatives in 25 mM HEPES at pH 7.4, not shown).

Since the binding of proteins to the QD surface may be strongly dependent on buffer composition and ionic strength, gel filtration separations were also performed in 100 mM phosphate as well as 25 mM HEPES with 50 mM NaCl added. For these results, see the Supporting Information and Figures S13 and S14.

In summary, gel filtration indicated weak or no complex formation between QD510 and all tested CytC derivatives. Some complex formation was detected between QD750 and Fe(III)CytC or ZnCytC.

#### FCS Analysis of QD–CytC Mixtures

Due to the fluorescence of QDs and CytC derivatives, the FCS technique could be used as a noninvasive alternative to gel filtration as a method to determine the formation and size of potential QD–CytC complexes. The limitation of FCS is a low concentration of fluorophores during measurements, which prevents the observation of processes with relatively high dissociation constants. FCS and gel filtration need to be considered as complementary methods. The hydrodynamic radii of fluorescent particles in a solution of QD–CytC mixtures were calculated based on the diffusion times obtained from autocorrelation analysis. The attempts at cross-correlation measurements failed due to leakage of QD510 fluorescence into the CytC channel after emission beam splitting or overlapping of the QD750 and CytC emission spectra. The autocorrelation analysis of pure QDs showed an increase in nanocrystal diameters related to their emission wavelength and demonstrated the homogeneity of colloidal solutions, excluding the possibility of spontaneous aggregate formation (Figure S15). Minor differences in hydrodynamic radii were detected for different fluorescent CytC proteins, which suggests some impact of iron ion removal or substitution on the protein structure.

**Figure 7:**
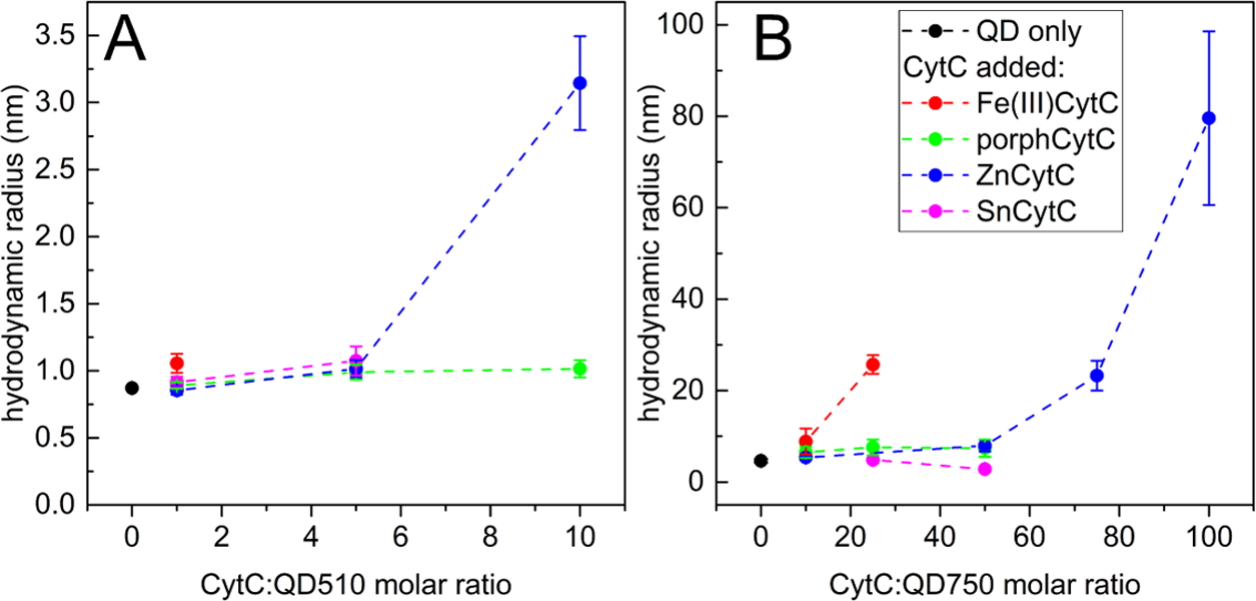
Hydrodynamic radii calculated from FCS measurements of QD and CytC mixtures. Different species of CytC protein were added in a given molar ratio to 0.3 μM QD510 (A) or 0.1 μM QD750 (B) in 25 mM HEPES at pH 7.5. Samples that were excited at 488 and 495–555 nm of BP or 505 nm of LP were used as emission filters for QD510 and QD750, respectively. Error bars represent standard deviations of six measurement repetitions on the same sample.

The hydrodynamic radii of QD510 (0.87 nm) and QD750 (4.62 nm) particles increased in the presence of CytC proteins, as determined by the autocorrelation results for QD fluorescence (Figure 7). The largest changes were for ZnCytC (3.14 nm for the 10:1 mix with QD510 and 79.6 nm for the 100:1 mix with QD750). A significant increase in the QD750 radius was also induced by a 25:1 molar excess of Fe(III)CytC (25.7 nm). This inconsistency with gel filtration results was also reported in a previous paper.^12^ As was proposed, it may result from the stacking of Fe(III)CytC complexes in the column or from the interactions with beads disturbing the weak binding of proteins to the QD surface.

In the cases of QD750 and ZnCytC, the radius of the complex depended on the increasing concentration of ZnCytC. This indicates that the surface of the large QD750 particle can bind more than one molecule of protein. The data in Figure 7 are not complete for every CytC species due to the very efficient quenching of QD510 (Fe(III)CytC and SnCytC) and the CytC-induced formation of large QD750 aggregates of immeasurable radius (Fe(III)CytC). A closer examination of FCS parameters, namely, *N*_p_ (average number of particles in confocal volume) and molecular brightness represented by cpp (number of counts of detected photons per particle; Figure S16), revealed the initiation of the aggregation process for the highest concentrations of ZnCytC (10:1 for QD510 and 100:1 for QD750). An increase in cpp and a parallel decrease in the *N*_p_ value indicate the presence of highly bright aggregates assembled from single particles and detected as one entity, reducing the apparent number of particles in focal volume.

Our study indicates that porphCytC and SnCytC may also bind to the QD surface, but in a weaker way than ZnCytC. This result seems to be in contradiction with gel filtration experiments, showing no bindings for these two CytCs. This may be explained explicitly by the weak binding, which is easily broken by friction on the column. The change in metal occupancy of the CytC porphyrin moiety seems to substantially affect the ability of the protein to bind to the QD surface (Table 2). Porphyrin ring occupancy might influence the CytC polypeptide chain very slightly,^56^ and the possible structure perturbation may not be sufficient to affect the interaction of this protein with the QD surface. There, the change of the central atom in the porphyrin moiety most likely influences the charge distribution over the CytC protein surface, indirectly changing the QD–CytC complex stability. ZnCytC and SnCytC are involved differently in the coordination sphere, as they need to be 5th-coordinated, while Fe is 6th-coordinated. A metal-free porphyrin ring, additionally, is no longer planar, which also changes the organization of amino acids in the heme neighborhood.

**Table 2:** Comparison of QD–CytC Binding Determination by Different Assays Used in the Study^a^

	CytC form	gel filtration	FCS	BLI assay	agarose gel electrophoresis
QD510	Fe(III)CytC	–	–	+	–
	porphCytC	–	–	+ (weakest)	–
	ZnCytC	+/–	+	+ (strongest)	+
	SnCytC	–	–	+	–
QD750	Fe(III)CytC	+	+		–
	porphCytC	–	–		–
	ZnCytC	+	+		+
	SnCytC	–	–		–

a The binding (+) or lack of it (−) are depicted; the valuable BLI experiment data for QD750 was not obtained. All of the experiments were performed in 25 mM HEPES at pH 7.4 (with the addition of 50 mM NaCl in the gel filtration buffer). For the agarose gel electrophorograms, see Figure S18.

As we observed, the assemblies of QD and CytC are shown to exhibit different stoichiometries, from possible 1:1 complexes to large aggregates, and to depend on the CytC:QD molar ratio and ionic strength of the buffer. A previous paper showed discrepancies between the assumption of simple attraction of oppositely charged QD and protein surfaces and the results of QD–CytC and QD–ferredoxin binding experiments.^12^ The nature of QD–CytC binding is certainly distinct from the canonical “lock and key” model of biomacromolecule interactions and creates ambiguity in using the term “specificity” in its description. To gain more insights into the direct interaction of CytC with QDs, we performed biolayer interferometry experiments (BLI) on QD510 and all CytCs (Figure S17). The different conditions of the assay were trialed to find a balance between mild enough to enable noncovalent loading of QD (to maintain the QD surface unchanged) and stringent enough to avoid nonspecific binding of CytC proteins to the sensor surface. The results of the so-compromised assay created difficulties in evaluating binding parameters, such as the affinity constant. In all cases, binding was detected, however, with a high rate of unbinding events. This indicates weak interactions, with no stable complex formation. Without the possibility of clearly calculating the binding constant, we can only speculate about weaker and stronger complexes from the set of collected data. Nevertheless, we were still able to obtain data to help answer our main questions.

## Conclusions

To conclude, our set of data suggests that both photoinduced ET and RET (by either the Förster or Dexter mechanism) from one QD donor may coexist if appropriate acceptors are present. It is due to irreversibility of Fe(III)CytC reduction in our system and the domination of ET effects, including irreversible wear of donors. In a holistic description, this may be seen as a preference for ET over RET. The results also suggest that there may be a cross-link between ET and RET, most probably electrons from the CB of QDs. This interaction is more likely synergistic, as we observed signs of a rate increase in the photoreduction experiment. The only observed inhibition (ZnCytC) was related to stable complex formation, blocking access to the QD surface. The presence of fluorescent CytC derivatives only slightly influences static quenching but increases the dynamic quenching efficiency, which suggests that the “interaction spots on the QD” surface are available in excess. This may mean either that the real QD surface area is large enough or that the exchange rate at the QD surface is fast enough, making it accessible. The nature of synergistic action observed in other cases may allow the hypothesis that RET by either the Förster or Dexter mechanism may be an additional way to power up the ET. In general, our findings indicate that any QD-like particles, applied in more complicated assays (including cells in the end), need to be considered as donors of electrons and energy at the same time. We also provide the basic framework for analysis of such systems.

## Supporting Information Available

Experimental details of fluorescence data fitting, FCS analysis, BLI and flash photolysis methods, statistical analysis; absorption and emission characteristics of QDs and CytCs used in the study and analysis of their statistical significance; results of Fe(III)CytC reduction experiments; detailed description of the procedure for calculation of quenching constants based on titration results; transient absorption decays; example of steady-state and time-resolved QD fluorescence measurements under CytCs titration; temperature dependence of quenching; Stern–Volmer plots; percentile contribution of individual exponential lifetime components for titration experiments; description of the theoretical model for QD quenching by mixtures of CytC derivatives; results of gel filtration experiments; hydrodynamic radii and cpp parameters calculated for FCS experiments; example of agarose electrophoresis results (PDF)
