# Supplementary material for: Competition between Photoinduced Electron Transfer and Resonance Energy Transfer in an Example of Substituted Cytochrome c–Quantum Dot Systems
Source: J Phys Chem B. 2021 Mar 24;125(13):3307–20. doi: 10.1021/acs.jpcb.1c00325 (PMC8041302; doi:10.1021/acs.jpcb.1c00325)
Supplement: Supplementary file 1 — jp1c00325_si_001.pdf [file jp1c00325_si_001.pdf]

Supplementary materials

Competition between Photoinduced Electron Transfer and Resonance Energy Transfer in an Example of Substituted Cytochrome c–Quantum Dot Systems

Jakub Sławski,[1] Rafał Białek,[2] Gotard Burdziński,[2] Krzysztof Gibasiewicz,[2] Remigiusz Worch[3] and Joanna Grzyb*[1]

[1]Department of Biophysics, Faculty of Biotechnology, University of Wrocław, ul. F. Joliot-Curie 14a, 50-383 Wrocław, Poland

[2]Faculty of Physics, Adam Mickiewicz University in Poznań, ul. Uniwersytetu Poznańskiego 2, 61-614 Poznań, Poland

[3]Institute of Physics, Polish Academy of Sciences, Al. Lotników 32/46, 02-668 Warsaw, Poland

*Joanna.grzyb@uwr.edu.pl

Experimentals

1. Fluorescence data fitting

Fluorescence decays were fitted using OriginPro 2015 (OriginLab Corporation, USA) with a multicomponent exponential decay equation:

$$I = I_0 + \sum_{i=1}^{n} A_i exp\left(\frac{-(t-t_0)}{\tau_i}\right),$$

where I is the recorded intensity, I_0 is the intensity offset, t is the time of measurement with t_0 time offset, A_i is the amplitude of the ith component, τ_i is the lifetime of the ith component and n is the total number of decay components. The satisfactory fits were obtained for n = 2 for fluorescent CytC derivatives and n = 3 for QDs.

The average fluorescence lifetimes (τ_{av}) were calculated using the amplitude-weighted mean:

$$\tau_{av} = \frac{\sum_{i=1}^{n} A_i \tau_i}{\sum_{i=1}^{n} A_i}.$$

Plots of fluorescence intensity and lifetime as functions of the quencher concentration obtained from titration experiments were transformed to the Stern–Volmer F_0/F and τ_{av0}/τ_{av} plots, where F_0 and τ_{av0} are fluorescence intensity and average lifetime in the absence of quencher and F and τ_{av} – in the given concentration of quencher. The Stern–Volmer plots were used to calculate the values of quenching constants. In the case of equal slopes of F_0/F and τ_{av0}/τ_{av} plots, the plots were fitted to the dynamic quenching equation:

$$F_0/F = \tau_{av0}/\tau_{av} = 1 + K_{SV}[Q],$$

where [Q] is the molar quencher concentration and K_{SV} is the dynamic (Stern–Volmer) quenching constant. When the value of F_0/F slope was greater than τ_{av0}/τ_{av} line slope, K_{SV} was calculated on the

basis of τ_{av0}/τ_{av} plot. Then, F_0/F plots were fitted to the equation for the combined (dynamic and static) mechanism of quenching, with a fixed value of K_{SV}:

$$F_0/F = (1 + K_{SV}[Q])(1 + K_a[Q]^n) = (\tau_{av0}/\tau_{av})(1 + K_a[Q]^n),$$

where K_a is the static quenching constant, and n is the theoretical number of binding sites for the quencher on the fluorophore surface.

2. FCS measurements

Alexa Fluor 488 dye (A488; ThermoFisher) with a diffusion coefficient of 414 μm^{2}s^{-1} at 25 °C in water[1] was used before the use of every cover slide for calibration of confocal volume after correction of the A488 diffusion coefficient for the temperature dependence of the water viscosity. For fitting the obtained fluorescence intensity data, the intensity autocorrelation function for isotropic three-dimensional diffusion, including intersystem crossing (triplet state of fluorophore), was used:

$$G(t) = 1 + \left(1 + \frac{f_T}{1-f_T}\exp\left(\frac{-t}{\tau_T}\right)\right) \times \frac{1}{N_p} \times \frac{1}{\left(1+\frac{t}{\tau_D}\right)\left(1+\frac{t}{\kappa^2\tau_D}\right)^{1/2}},$$

where t is the lag time, f_T is the fraction of fluorophores in the triplet state with lifetime τ_T, N_p is the average number of fluorescent particles in the focal volume, τ_D is the diffusion time of investigated particles and s is the so-called structural parameter equal $\kappa = z_0 / w_0$ (where w_0 and z_0 are, respectively, the lateral and axial radii of the confocal volume $\times^{-2}$). Diffusion coefficients were calculated based on the diffusion times obtained from data fitting:

$$D = \frac{w_0^2}{4\tau_D},$$

and then the hydrodynamic radii r were determined using the Einstein–Stokes equation:

$$r = \frac{k_B T}{6\pi\eta D},$$

where k_B is the Boltzmann constant (1.38×10^{-23} J/K), T is the absolute temperature during measurements and η is the water viscosity at temperature T. cpp (counts per particle) parameters were calculated based on the relationship:

$$cpp = \frac{\nu_C}{N_P} = \nu_C(G(0) - 1),$$

where ν_C is the rate of photon counts expressed in Hz, and $G(0)$ is the value of the intensity correlation function in time $t = 0$.

The concentrations of QDs were 0.3 μM for QD510 and 0.1 μM for QD550 and QD750 in 25 mM HEPES at pH 7.5. The CytC proteins were added in a 1:1 to1:100 CytC:QD molar ratio.

3. Bio-layer interferometry assay

Binding parameters of QD510-CytC pairs obtained by bio-layer interferometry (BLI) experiments using the Octet (ForteBio) instrument. The assays were performed at 25 °C. Nickel-nitrilotriacetic acid (NTA) sensors were coated during the assay with His-tag-mCherry protein (500 sec of incubation, 1 μM in 25 mM Tris, 500 mM NaCl and 10% glycerol at pH 7.4), which exhibits high affinity to both nickel ions and QDs and was used as a linker to attach QD510 to the sensors. Next, QD510 was immobilised (500 sec, 2 μM in 25 mM Tris, 500 mM NaCl and 10% glycerol at pH 7.4) on the sensors. The association of CytC derivatives (1 μM) was monitored in 25 mM HEPES at pH 7.4, followed by dissociation in the same buffer (500 sec and 600 sec, respectively). The constants were obtained from fitting of binding curves to the 1:1 binding model after subtraction of the reference data (sensors without QD510 loading were used as a control for the unspecific binding of CytC proteins to the sensor surface).

4. Flash photolysis

The transient absorption system was designed as described earlier.[2] Briefly, pump pulses (355 nm, 8 ns FWHM, 0.5 Hz repetition rate, 1 mJ) were generated by a Q-switched Nd:YAG laser (Continuum Surelite II). As the probe, a 150-W xenon arc lamp (Applied Photophysics, UK) was used either in pulsed (for time window <500 μs) or steady-current mode (for time window >500 μs). The probe passed through the sample with a 1 Hz repetition rate obtained using a shutter (Uniblitz). A monochromator (Acton Research Spectra Pro 300i) was used to disperse the probe light, and a photomultiplier (R928 Hamamatsu) coupled with a digital oscilloscope (Tektronix TDS 680 C) was used for detection. Samples (1 ml) were placed in a quartz cuvette (10 mm x 10 mm cross section). Measurements were performed at room temperature in 25 mM HEPES at pH 7.4 and deoxygenated with argon for at least 15 min. The concentrations of samples were adjusted to yield at least 0.4 absorbance at the excitation wavelength (355 nm), and they were 1 μM QD630, 25 μM Fe(III)CytC, 15 μM ZnCytC and 0.5 μM QD630 + 5 μM Fe(III)CytC when these two were mixed.

Table S1 Physicochemical properties of QDs and CytC derivatives used in experiments.

QDs	emission maximum λ_{em} [nm]	first excitonic peak λ_{exc} [nm]	extinction coefficient for λ_{exc} [$M^{-1}cm^{-1}$]	diameter [nm]	midpoint potential (E_m vs NHE) [mV]
QD510	507	476	24 626.7	1.53	-970§
QD550	546	508	73 064.2	2.55	-770
QD630	627	598	155 875.2	3.64	-680§
QD750	760	~700	450 274.2	6.01	-490

CytC form	absorption maxima [nm]	extinction coefficient for Soret peak [$mM^{-1}cm^{-1}$]	emission maxima [nm]	midpoint potential (E_m vs NHE) [mV]
Fe(III)CytC	409, 530	106.1	-	+286
Fe(II)CytC	414, 518, 548	129.1	-	+286
porphCytC	397, 503, 538, 565, 618	81.0	615, 679	-
ZnCytC	414, 542, 578	243.0	580, 632	-
SnCytC	403, 536, 574	267.0	574, 628	-

Spectral overlap integrals J [$nm^4 M^{-1}cm^{-1}$] and Förster radii R_0 [nm]

		Acceptors									
		FeIIICytC		FeIICytC		porphCytC		ZnCytC		SnCytC	
		J	R_0	*J*	R_0	*J*	R_0	*J*	R_0	*J*	R_0
Donors	QD 510	4.73e14	3.13	6.0e14	3.26	2.80e14	2.87	6.53e14	3.31	2.70e14	2.85
	QD 550	5.16e14	3.98	6.94e14	4.18	2.63e14	3.55	1.14e15	4.54	5.46e14	4.01

		Acceptors			
		QD630		QD750	
		J	R_0	*J*	R_0
Donors	porph CytC	9.54e15	4.10	1.21e17	6.27
	ZnCytC	1.385e16	3.52	1.242e17	5.08
	SnCytC	1.365e16	3.33	1.259e17	4.83

QDs diameters and extinction coefficients were calculated according to Yu et al[3]. The calculations are based on the wavelength of the first excitonic peak of absorption spectrum; in the case of QD750 the peak was weakly apparent and its position was approximated to 700 nm. Extinction coefficient of CytC derivatives were taken from Vanderkooi at al.[4] and Darżynkiewicz et al[5]. Midpoint potential of FeCytC from Gopal et al[6]. Midpoint potential of QDs based on Grzyb et al.[7], § - values estimated from other given in the paper. Spectral overlap integrals (J) calculated from original spectra with a|e - UV-Vis-IR Spectral Software 2.2, FluorTools, www.fluortools.com. R_0 calculated from J given here and QD quantum yield measured by relative method, with AlexaFluor 488 used as a standard (Φ=0.92)[8]. Φ of CytC derivatives from Vanderkooi et al.[4]

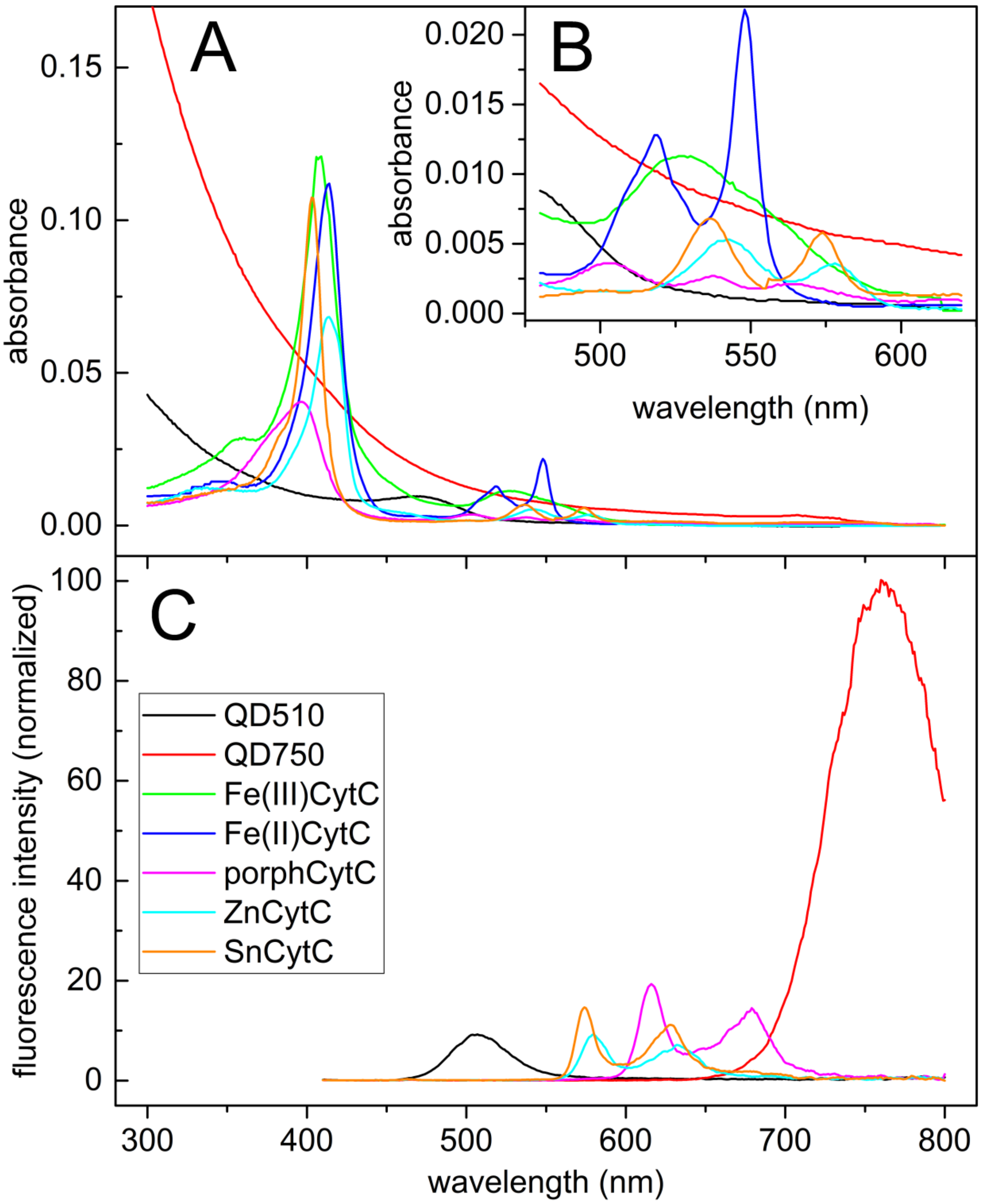

Fig. S1 Absorption (**A**, and inset with magnified 480-620 nm range, **B**) and emission (**C**) spectra of CytC proteins and quantum dots used in titration experiments. Spectra of CytC proteins (0.5 μM), Q510 (0.5 μM) and QD750 (0.01 μM) were measured in quartz cuvettes (10 mm optical pathway) in 25 mM HEPES pH 7.5 buffer. Excitation wavelength for emission spectra was 404 nm.

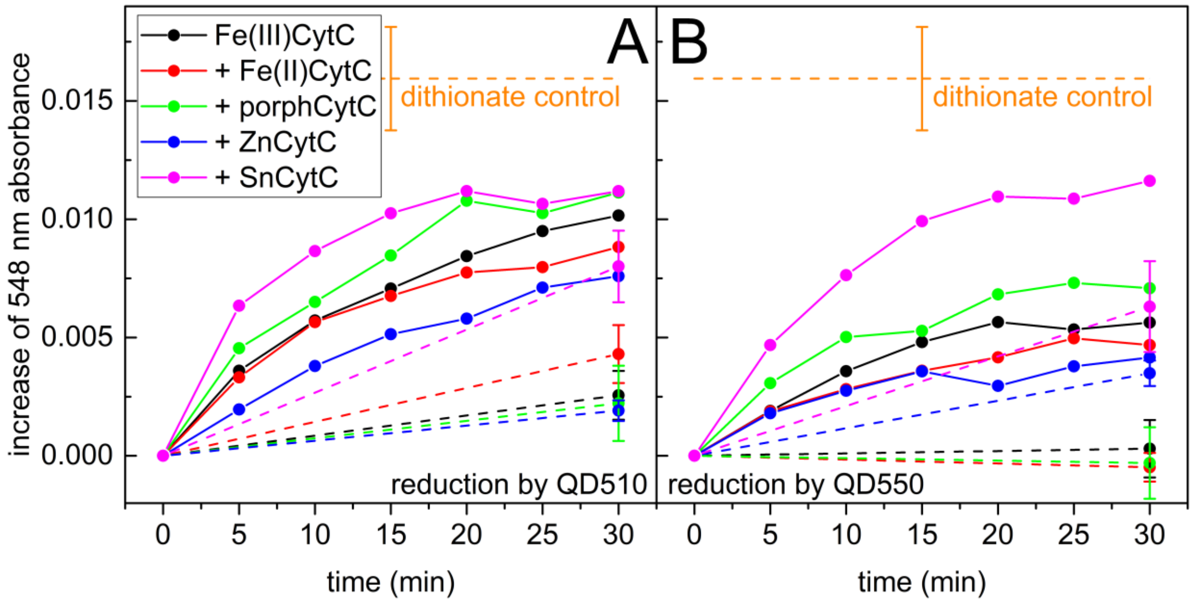

Fig. S2 The results of Fe(III)CytC reduction experiments. The absorbance at 548 nm was measured as indicator of Fe(III)CytC (1 µM in 25 mM HEPES pH 7.5) reduction induced by QD510 (0.1 µM) (**A**, solid lines) or QD550 (0.1 µM) (**B**, solid lines) illuminated by UV lamp through 300-400 nm band-pass filter. CytC derivatives were added in 1 µM concentration as indicated. The linear background (dashed lines) was created on the basis of initial time point and the reduction level in control samples dark-incubated for 30 min. The absorbance of dithionate-treated samples (orange dashed line) is shown. Error bars represent standard deviations of at least three independent repetitions, for clarity error bars for illuminated samples are omitted (shown on Fig.3, main manuscript).

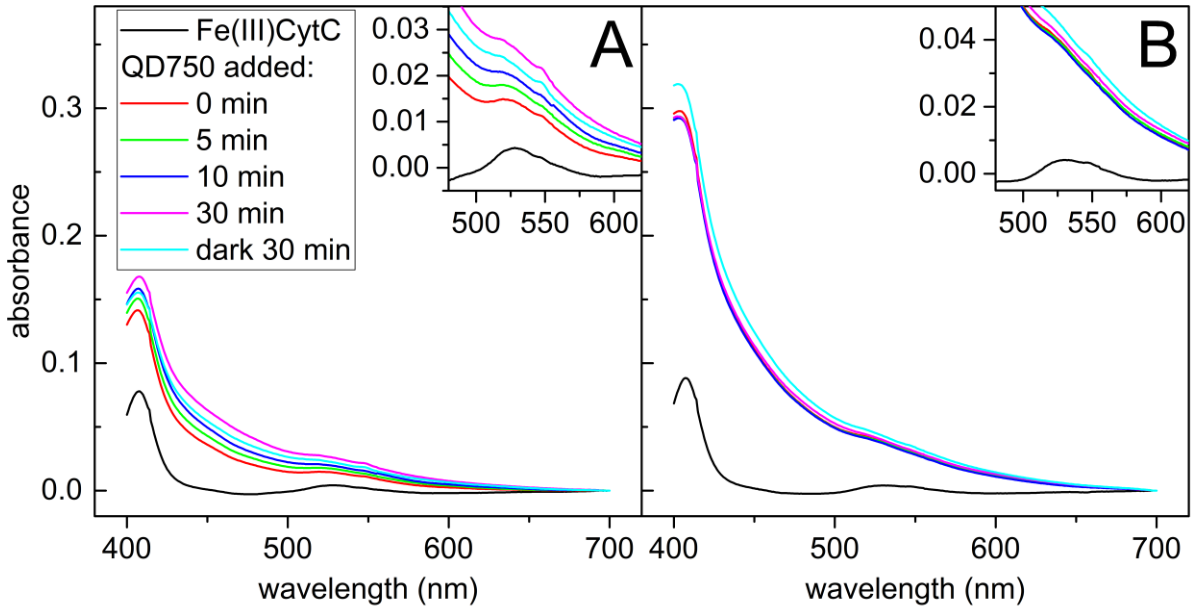

Fig. S3 Absorption changes during the UV illumination of 0.04 µM (**A**) or 0.01 µM (**B**) QD750 and 1 µM Fe(III)CytC. Insets show magnified spectra in 500-600 nm range. Samples were in 25 mM HEPES pH 7.5, illuminated through 300-400 nm band-pass filter.

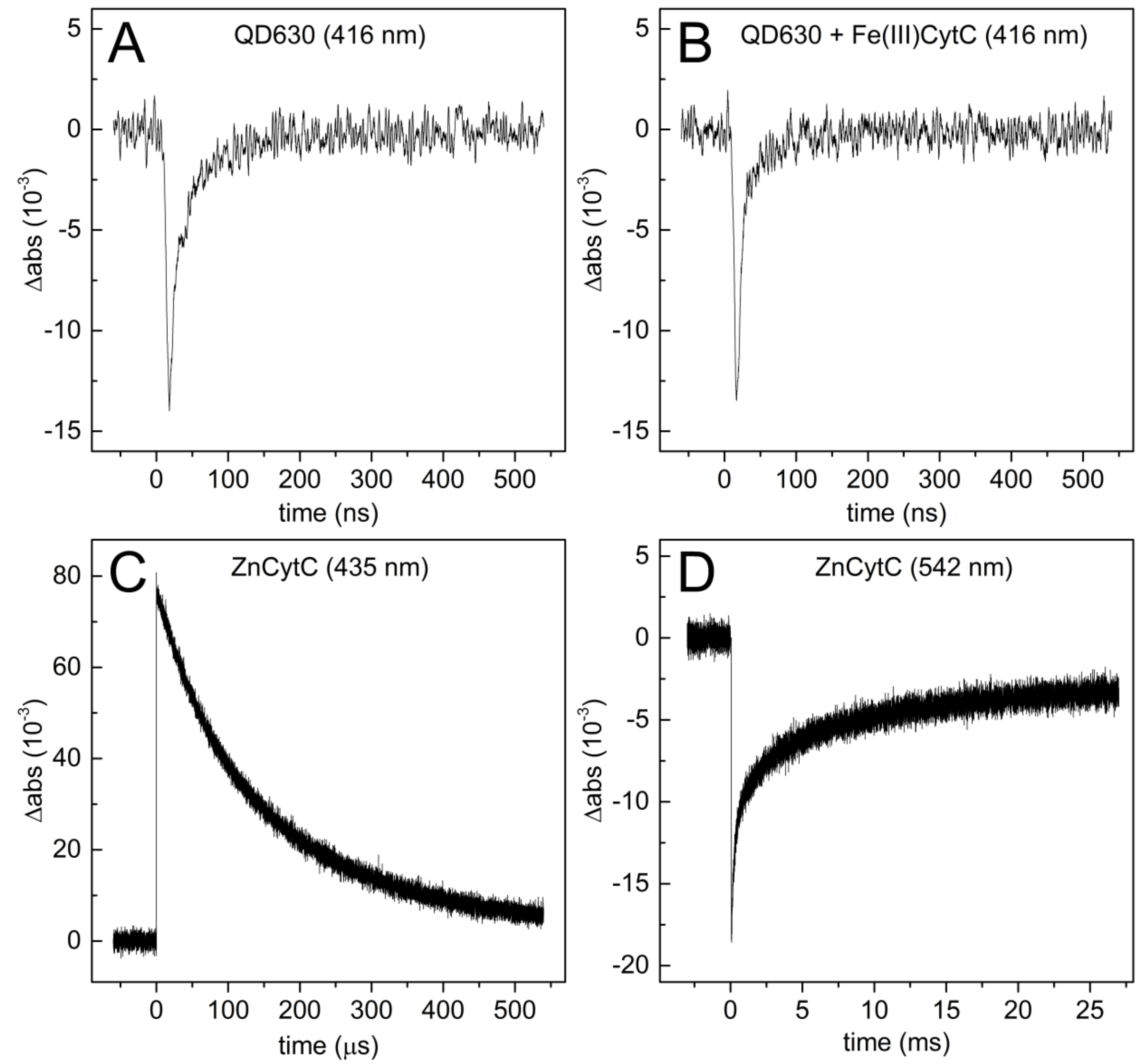

Fig. S4 Transient absorption decays recorded for 1 μM QD630 (A), 0.5 μM QD630 + 5 μM Fe(III)CytC (B) and 15 μM ZnCytC (C,D) in 25 mM HEPES, pH 7.4. Samples were excited by 355 nm pulses and transient absorption kinetics were probed at selected wavelengths (416, 435 and 542 nm).

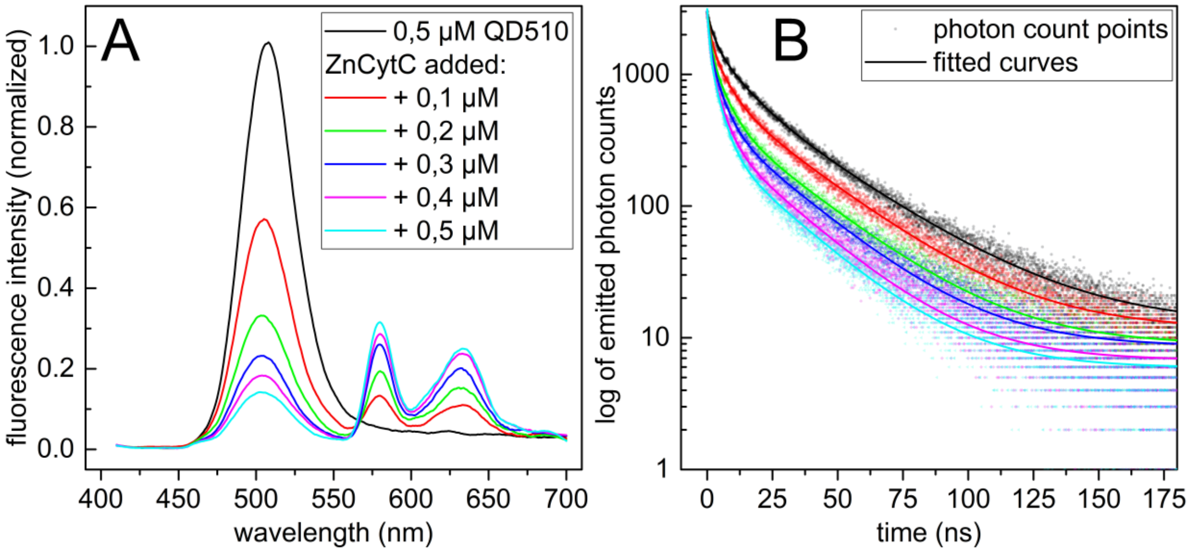

Fig. S5 The example of steady-state (**A**) and time-resolved (**B**) QD510 fluorescence measurements in the presence of increasing concentration of ZnCytC. QD510 (0.5 µM) solution in 25 mM HEPES, pH 7.5 was titrated with small volumes of ZnCytC solution. For every titration step (in the case of fluorescence intensity (**A**), every second measurement is shown for clarity) fluorescence was measured for λ_{ex} at 404 nm. For time-resolved fluorescence (**B**) λ_{ex} at 404 nm and λ_{em} at 510 nm. Solid lines on graph **B** represent curves fitted to experimental data (points), using three-exponential decay function.

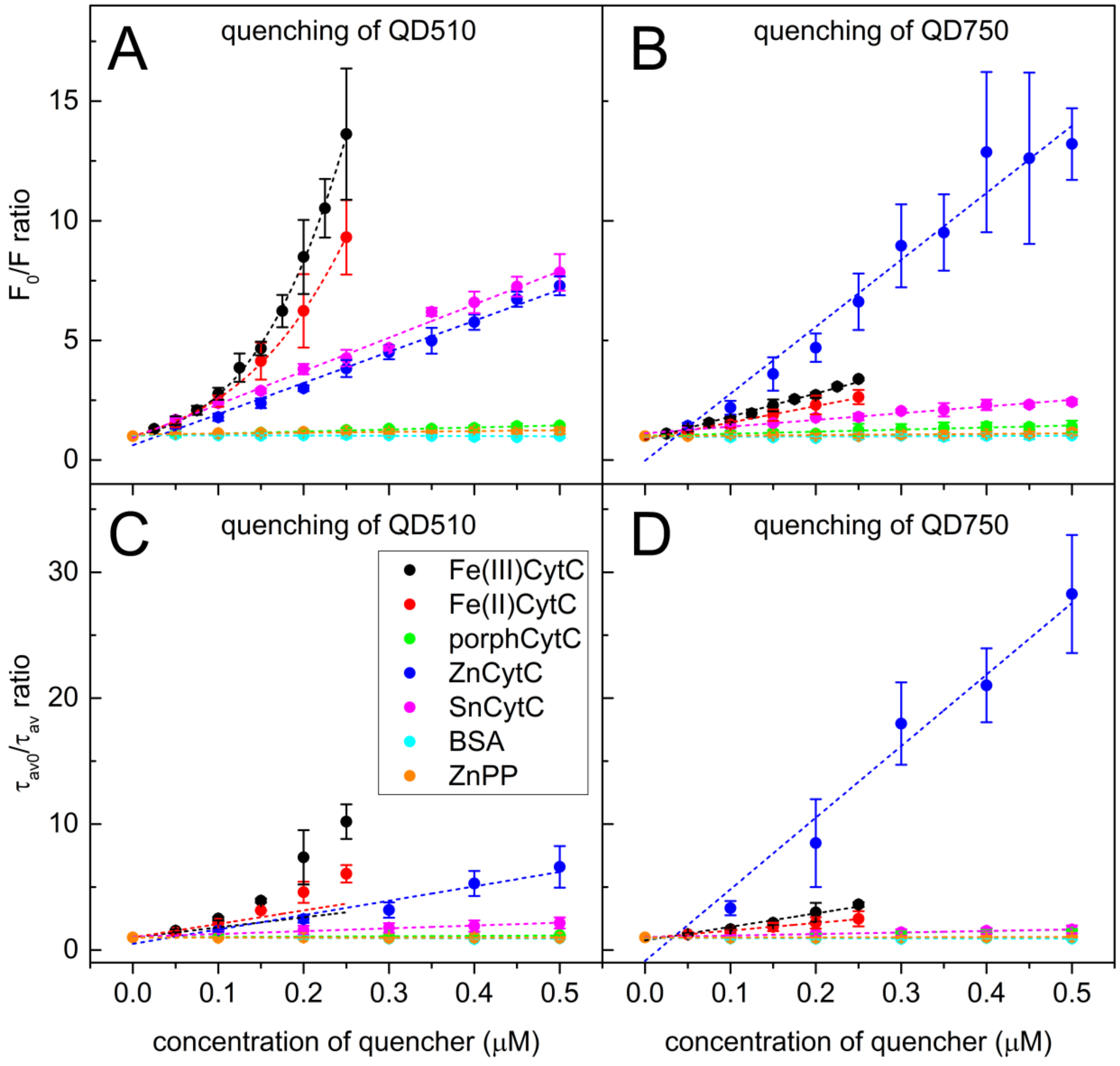

Fig. S6 Stern-Volmer plots of F_0/F (**A, B**) and τ_{av0}/τ_{av} (**C, D**) obtained by the titration of QD510 and QD750 (0.5 μM and 0.01 μM, respectively, in 25 mM HEPES, pH 7.5) by above-mentioned quenchers in concentration range 0 μM – 0.5 μM (0 μM – 0.25 μM for Fe(III)CytC and Fe(II)CytC – due to the excessive quenching in higher concentrations). The values of QD fluorescence intensity (F) and average lifetime (τ_{av}) in a given quencher concentration were divided by initial quencher-free values (F_0 and τ_{av0}) and reciprocals of calculated ratios were depicted on the plots. Error bars represent standard deviations of at least three independent repetitions. Dashed lines represent fits of data point to Stern-Volmer equations: to linear formula for dynamic quenchers or to polynomial formula for combined mechanism of quenching. Lines calculated for τ_{av0}/τ_{av} plots of QD510 titrations by Fe(III)CytC and Fe(II)CytC are tangents to initial part of curve (see note below).

Two abnormalities from Stern-Volmer theory should be noted here. First is upward curvature of some τ_{av0}/τ_{av} plots (for titrations of QD510 by Fe(III)CytC, Fe(II)CytC and ZnCytC) what is in contradiction to standard assumption of the linearity of these plots. The possible explanation of this bimodal curve behavior is the presence of at least two processes decreasing the lifetime of QDs. For K_{SV} derivation the tangent to initial τ_{av0}/τ_{av} plot was taken for fitting to dynamic quenching equation. Second anomaly is the slope value of τ_{av0}/τ_{av} curve for QD750 titration by ZnCytC exceeding ca. two times the slope value for F_0/F plot. For this case, pure dynamic quenching was assigned and F_0/F plot was taken for fitting and K_{SV} calculation.

The temperature dependence of QD510 quenching by CytC derivatives

Dynamic quenching is usually the result of transient interactions driven by thermal collisions of fluorophore and quencher particles. Hence, dynamic quenching efficiency increases in a temperature-dependent manner. The opposite is true for a static quenching: increasing temperature disturbs a molecular complex formation. The character of quenching, caused by CytC proteins on QD510 fluorescence, was also evaluated in the 10–40 C range of temperature (Fig. S7). Quenching efficiency of Fe(III)CytC, defined as F0/F ratio, decreases in higher temperatures, revealing a predominant static component. The effectiveness of QD510 quenching by ZnCytC and SnCytC increases with growing temperature, which indicates the significant dynamic component in their quenching mechanism. This is highlighted especially by the increasing τ_{av0}/τ_{av} ratio – the most potent dynamic quencher, ZnCytC, exhibits the largest temperature-dependent increase of this parameter.

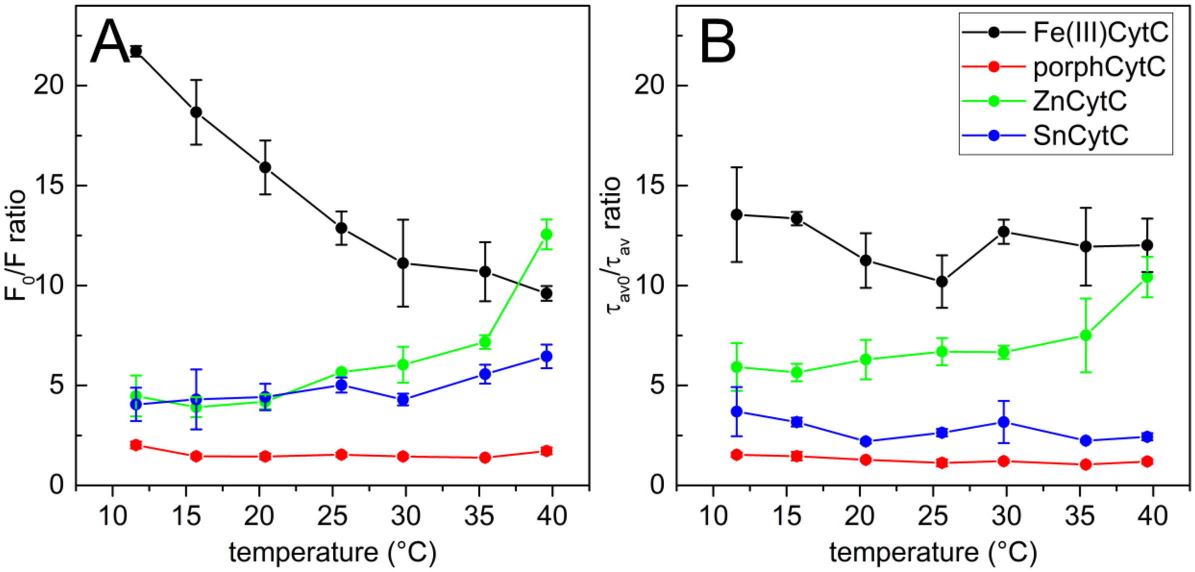

Fig. S7 Temperature dependence of QD510 quenching by CytC derivatives. The 0.5 μM QD510 fluorescence intensity and lifetime were measured before and after the addition of 0.5 μM CytC proteins. The obtained values are shown as F0/F (A) and τav0/τav (B) ratios. Experimental conditions and fluorescence measurement parameters are the same as in Fig. 4. Error bars represent standard deviations of at least three independent repetitions.

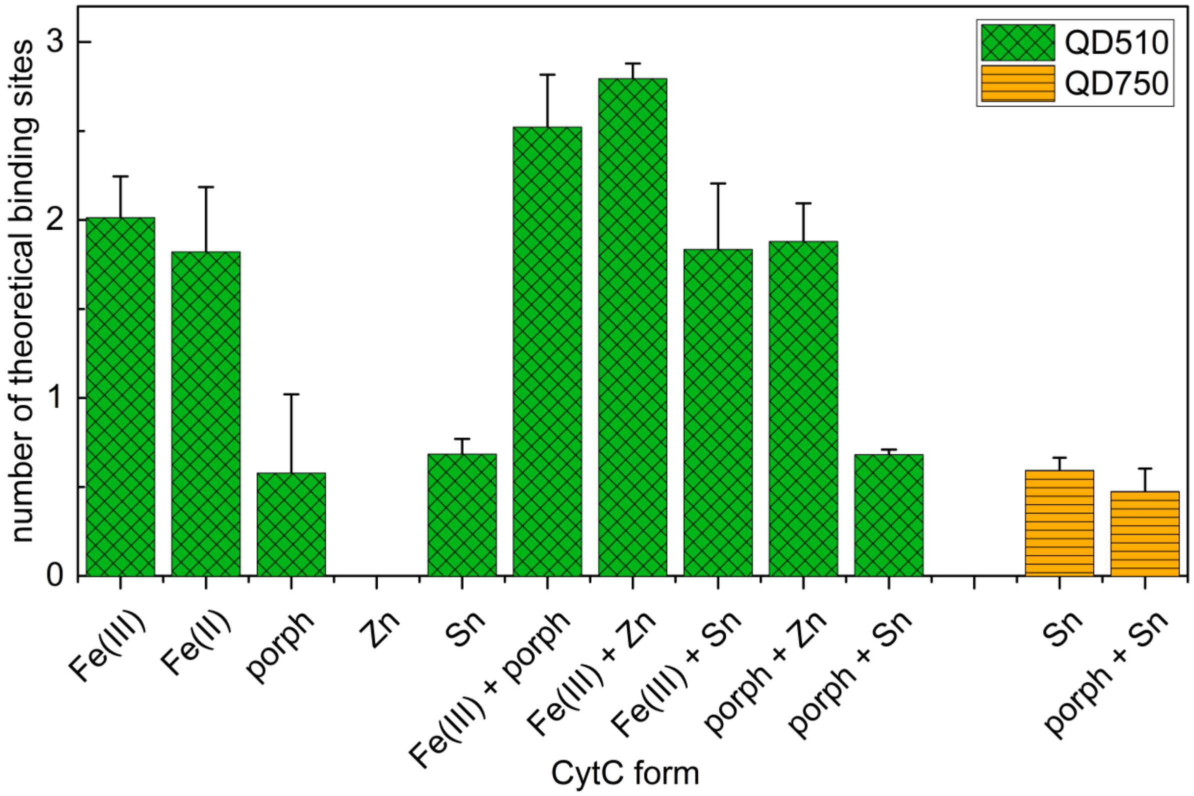

Fig. S8 The number of binding sites approximated from the fitting of QD titration results in the equation of the combined quenching mechanism. The single species of CytC or the mixtures of two different forms were used to titrate the QD solution. Experimental conditions and fluorescence measurement parameters are the same as in Fig. 4. Error bars represent standard deviations of at least three independent repetitions.

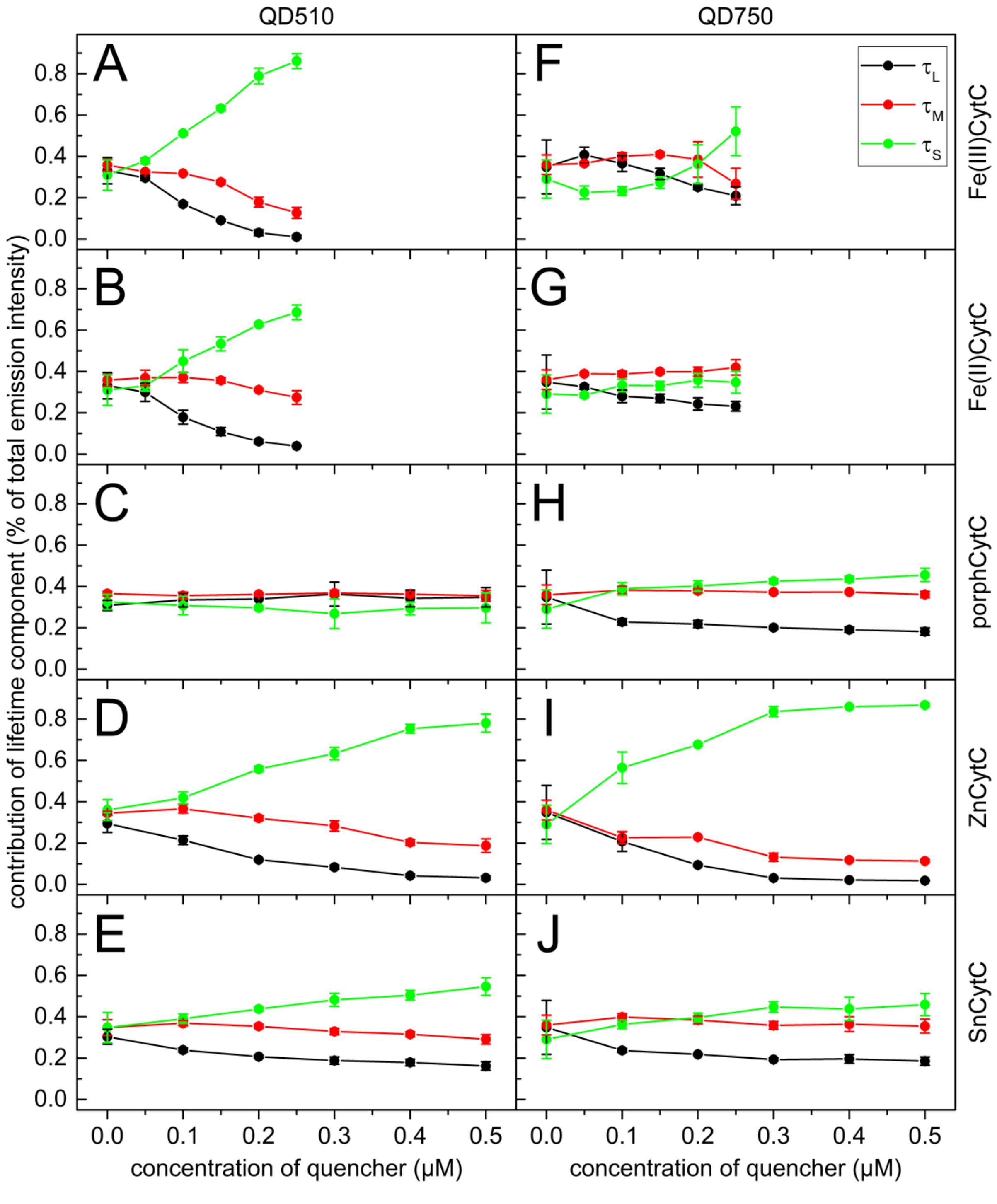

Fig. S9 The percentile contribution of individual exponential lifetime components for measurements obtained during the titration of QD510 (**A-E**) and QD750 (**F-J**) by the set of CytC species in concentration range 0 μM – 0.5 μM (0 μM – 0.25 μM for Fe(III)CytC and Fe(II)CytC – due to the excessive quenching in higher concentrations). The contribution is calculated on the basis of amplitudes (pre-exponential factors) of the respective components. τ_L, τ_M, and τ_S denote the longest, medium and the shortest component, respectively. Error bars represent standard deviations of at least three independent repetitions.

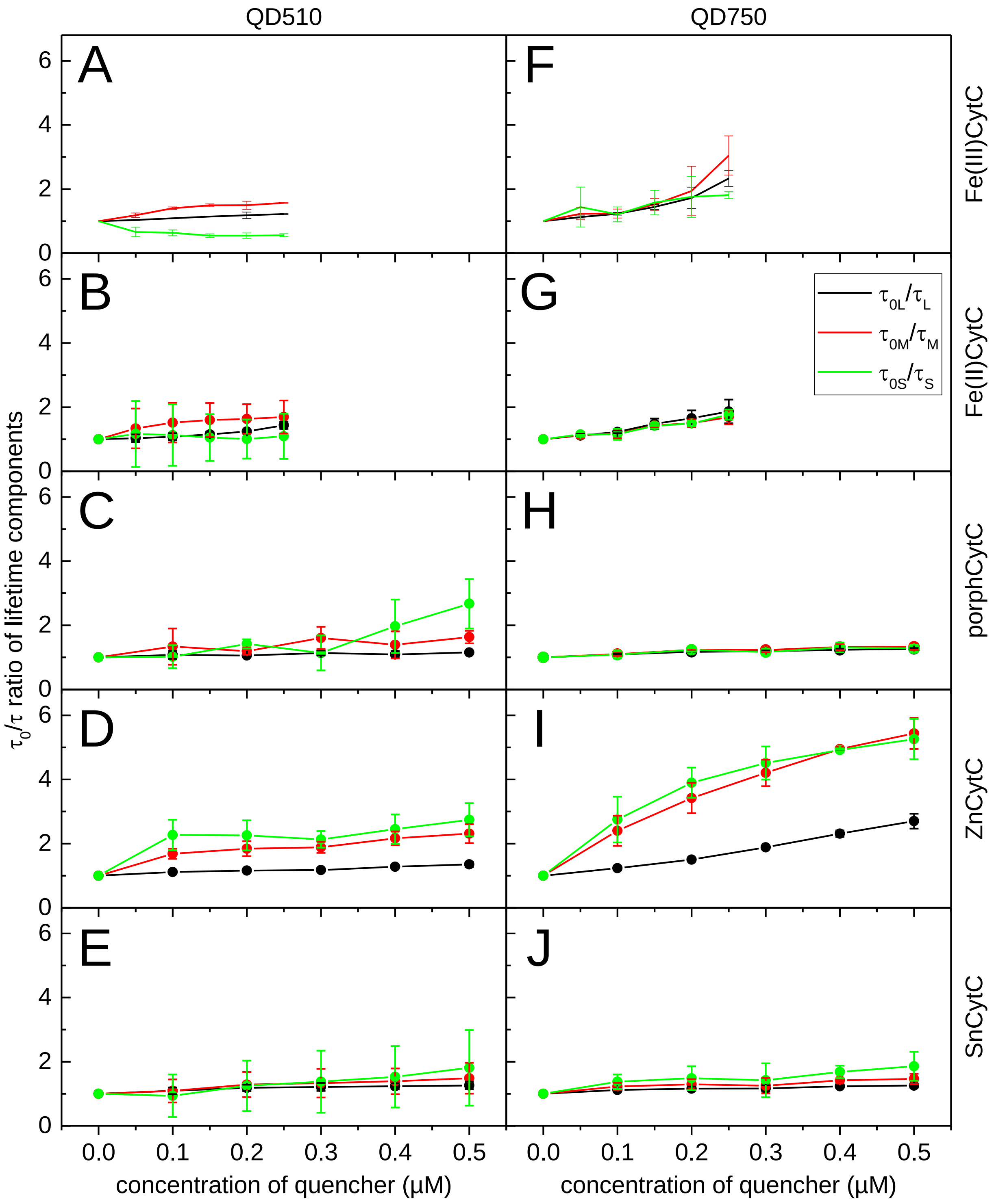

Fig. S10 The Stern-Volmer plots for individual exponential lifetime components for measurements obtained during the titration of QD510 (**A-E**) and QD750 (**F-J**) by the set of CytC species in concentration range 0 µM – 0.5 µM (0 µM – 0.25 µM for Fe(III)CytC and Fe(II)CytC – due to the excessive quenching in higher concentrations). τ_L, τ_M, and τ_S denote the longest, medium and the shortest component, respectively. Error bars represent standard deviations of at least three independent repetitions.

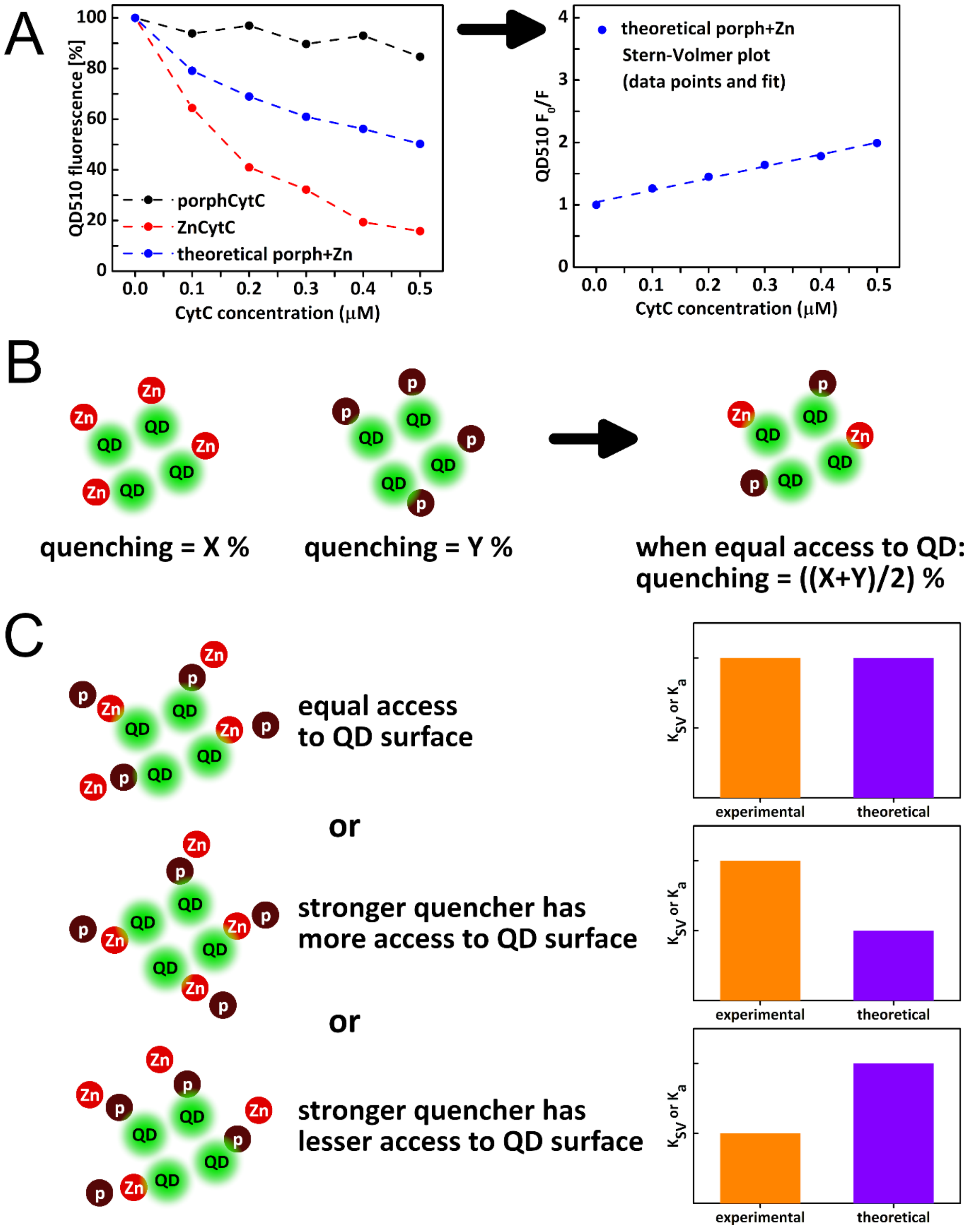

Fig. S11 Theoretical model for QD quenching by mixtures of CytC derivatives. Simulated fluorescence intensity and lifetime data were calculated as arithmetic mean of experimental points for two given CytC proteins (**A**, on example of QD510 fluorescence intensity in the presence of porphCytC and ZnCytC) and then fitted to appropriate Stern-Volmer equation. The assumption of theoretical model was equal access of quenchers (in equimolar concentrations) to QD (**B**). The comparison of theoretical and experimental quenching constants gives insight on competition processes between different CytC forms (**C**).

Construction of the simulated quenching constant

Simulated quenching constant, representing the constant expected for no interaction between ET and RET, was constructed by taking the average values of points of titration curves for two given CytC forms (creating the theoretical titration curves lying equidistantly between two titration curves for given single CytC forms). Such a method was based on the assumption that the mixture, containing two ideally non-interacting assays, may be virtually represented by two mixtures containing each assay separately. Then, if two cuvettes containing each of the assays were placed simultaneously in the light pathway, the recorded signals would serve (after correction for concentration) as titration curves. Such a measurement is easy to carry out in a transmission or absorption mode. However, due to the specific geometry of a fluorometric apparatus, it is impossible to ensure equal illumination of joined cuvettes, and the necessary values are obtained by mathematical transformation from two independent measurements. The simulated data were then fitted to Stern–Volmer equations to calculate quenching constants.

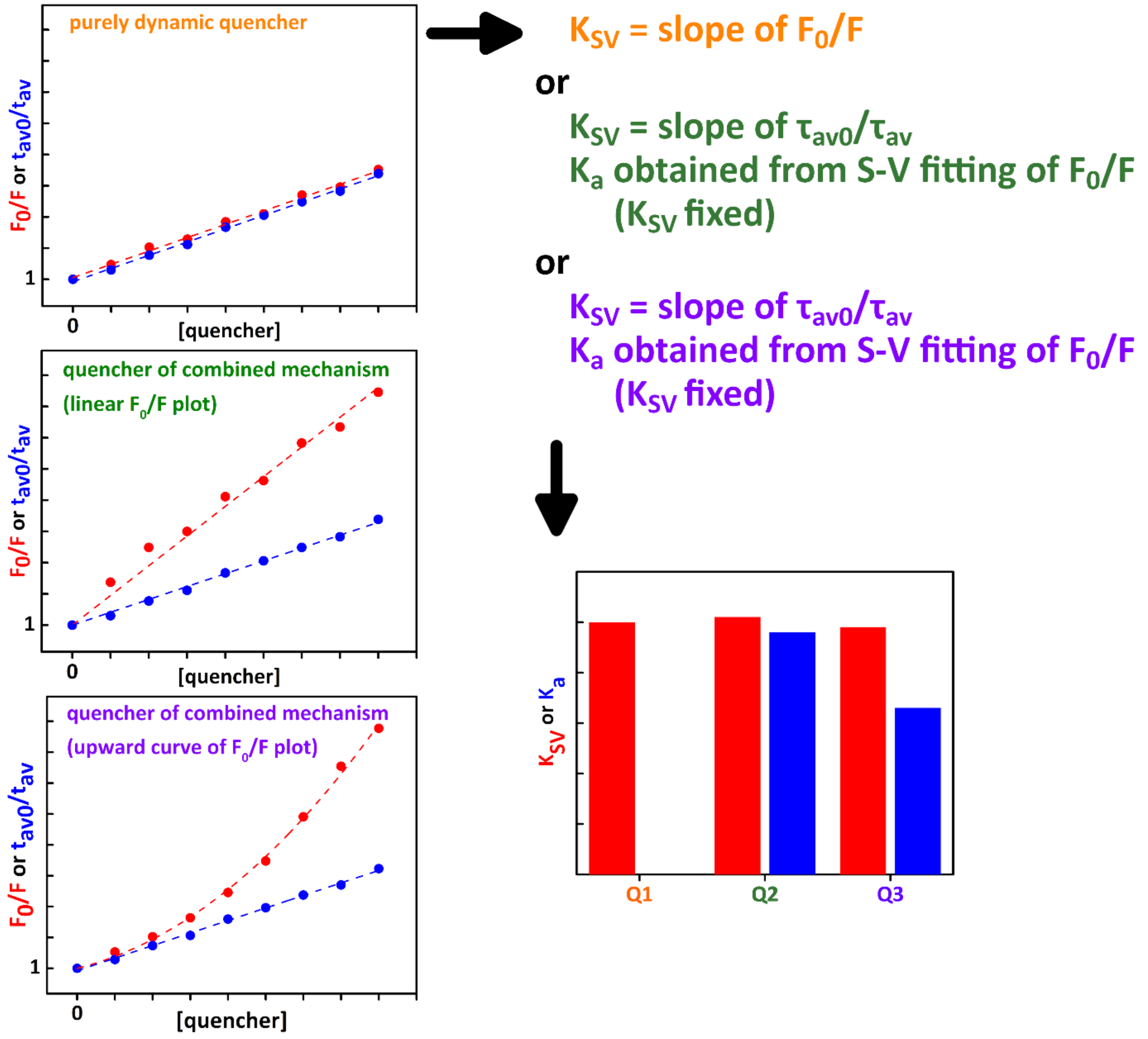

Fig. S12 Procedure for calculation of quenching constants based on titration results. Depending on the character of CytC quencher, K_{SV} value was obtained as a slope of F_0/F plot (for dynamic quenchers) or τ_{av0}/τ_{av} plot (for combined mechanism of quenching). Ka value for static component of quenching was calculated by fitting of F_0/F plot to Stern-Volmer equation for combined quenching mechanism (K_{SV} value obtained from τ_{av0}/τ_{av} slope was fixed the during the fitting).

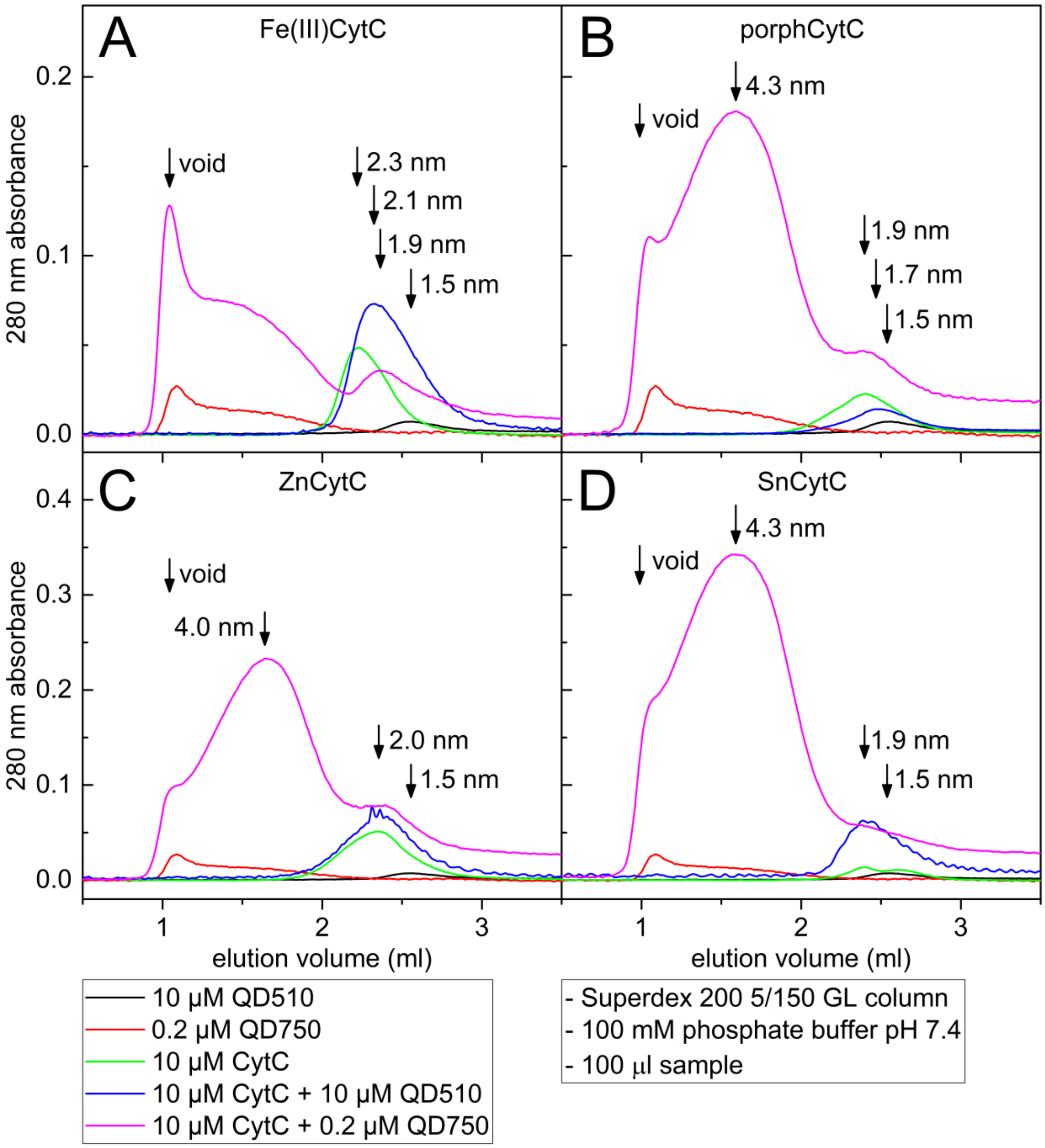

Fig. S13 Chromatograms of gel filtrations recorded for Fe(III)CytC (**A**), porphCytC (**B**), ZnCytC (**C**) and SnCytC (**D**). Each set of samples included only-QDs samples, only-CytC samples and QD-CytC mixtures. 100 μl samples were loaded on Superdex 200 5/150 GL column in 100 mM phosphate buffer, pH 7.4. Concentration of proteins was 10 μM in 1:1 (QD510) or 50:1 (QD750) molar ratio to the QDs. The hydrodynamic radii (or void volume of column) are depicted.

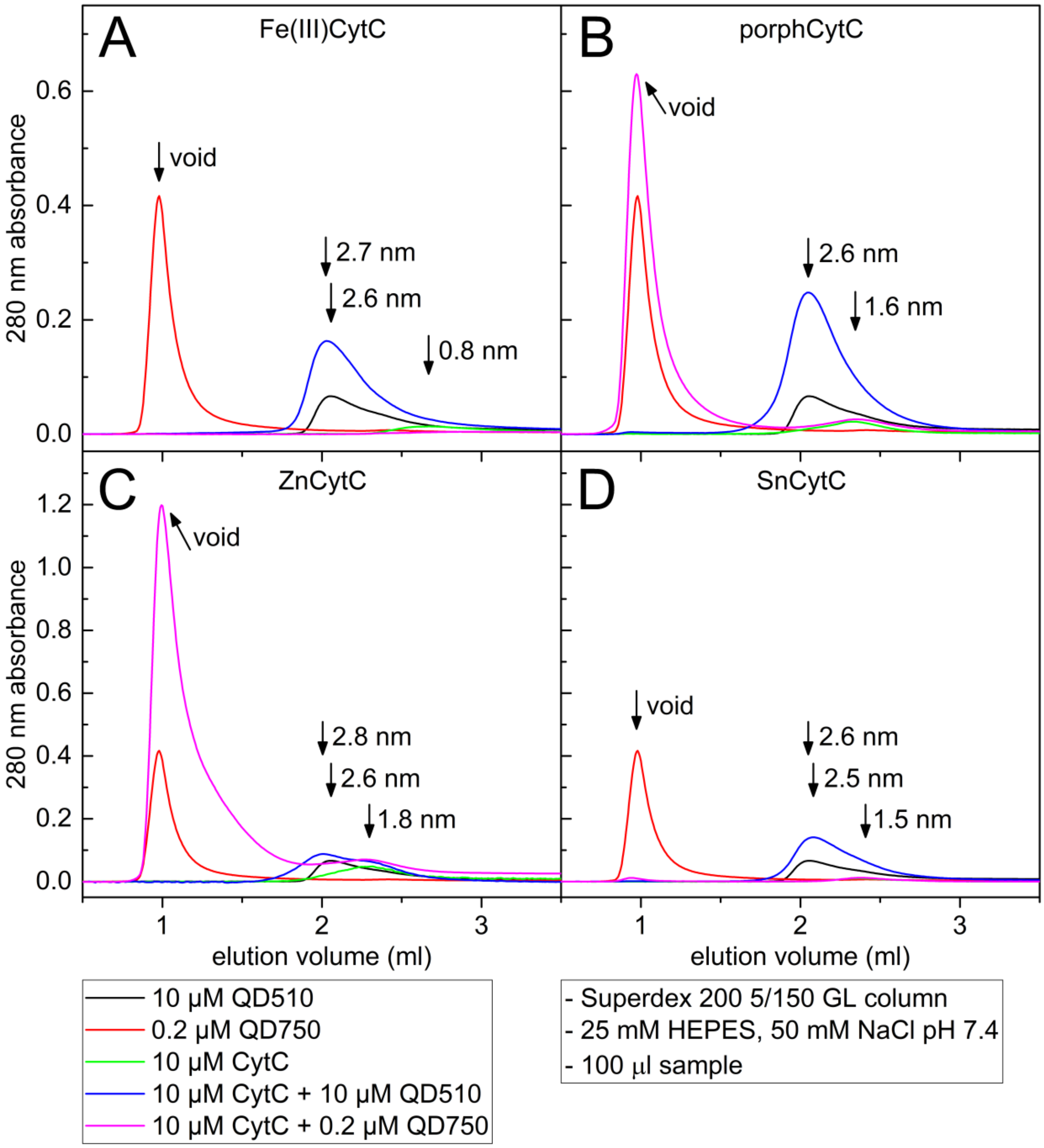

Fig. S14 Chromatograms of gel filtrations recorded for Fe(III)CytC (**A**), porphCytC (**B**), ZnCytC (**C**) and SnCytC (**D**). Each set of samples included only-QDs samples, only-CytC samples and QD-CytC mixtures. 100 µl samples were loaded on Superdex 200 5/150 GL column in 25 mM HEPES buffer, pH 7.4, with 50 mM NaCl. Concentration of proteins was 10 µM in 1:1 (QD510) or 50:1 (QD750) molar ratio to the QDs. The hydrodynamic radii (or void volume of column) are depicted.

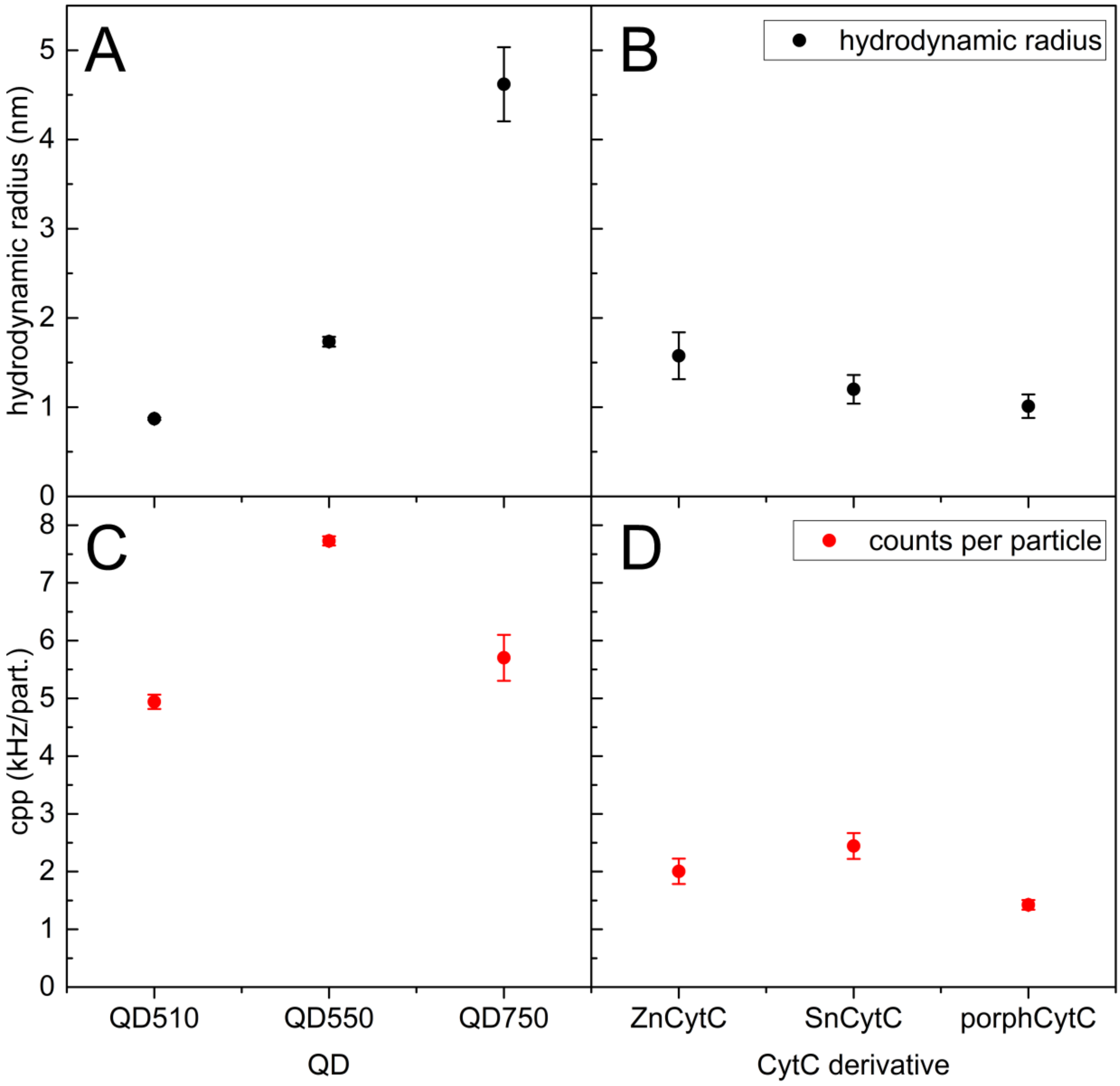

Fig. S15 Hydrodynamic radii (**A, B**) and cpp parameters (**C, D**) of QDs and fluorescent CytC proteins obtained by FCS measurements. The concentrations were: 0.3 µM for QD510 and 0.1 µM for other QDs and CytC. Samples were excited at 405 nm (CytC) or 488 nm (QDs) and 495-555 nm BP or 505 nm LP were used as emission filters for QD510 and others, respectively. Error bars represent standard deviations of six measurement repetitions on the same sample.

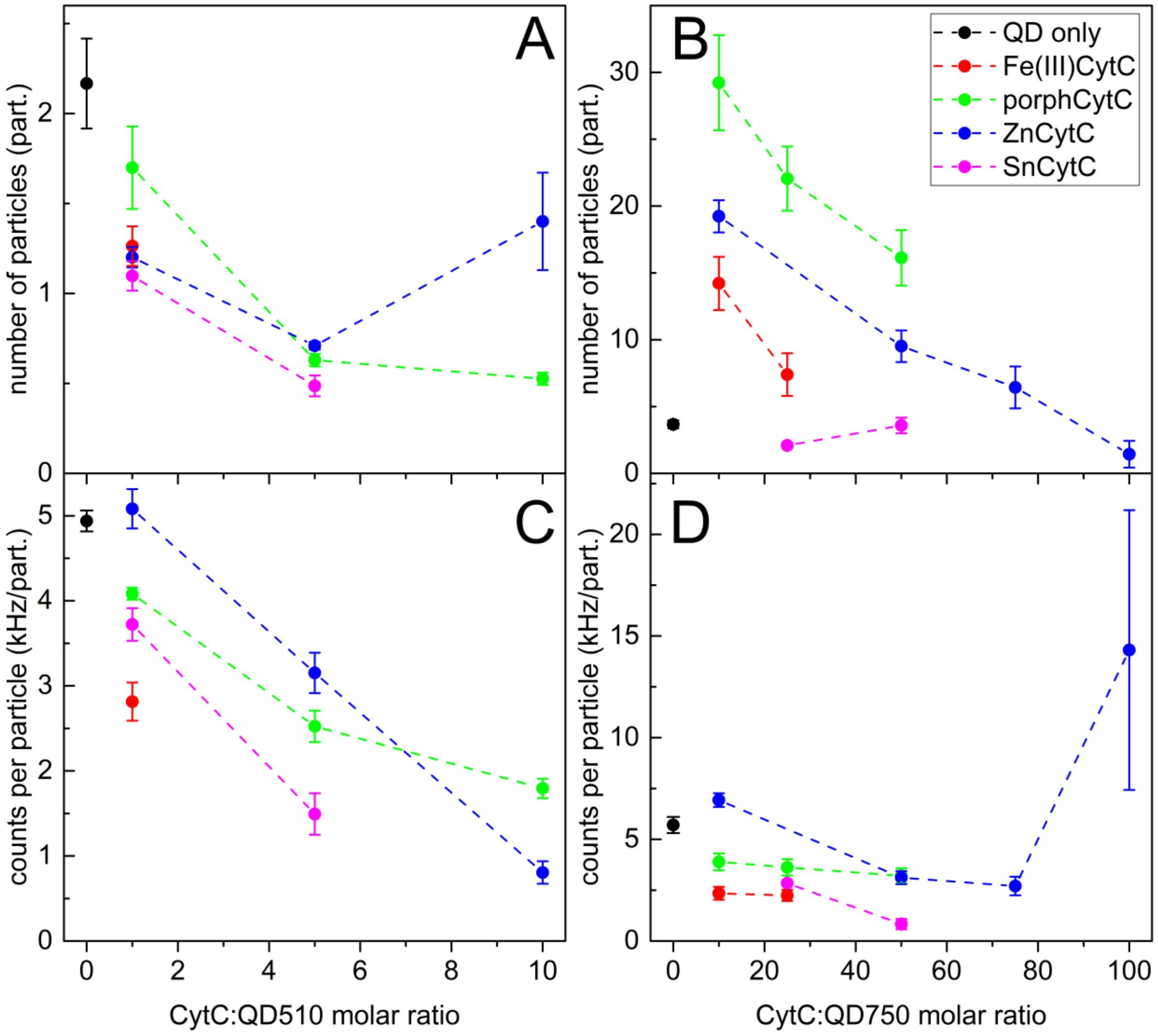

Fig. S16 Number of particles (**A, B**) and cpp parameters (**C,D**) of QD:CytC mixtures obtained by FCS measurements. The concentrations were: 0.3 µM QD510 and 0.1 µM QD750, CytC proteins were added in a given molar ratio. Samples were excited at 488 nm and 495-555 nm BP or 505 nm LP were used as emission filters for QD510 and QD750, respectively. Error bars represent standard deviations of six measurement repetitions on the same sample.

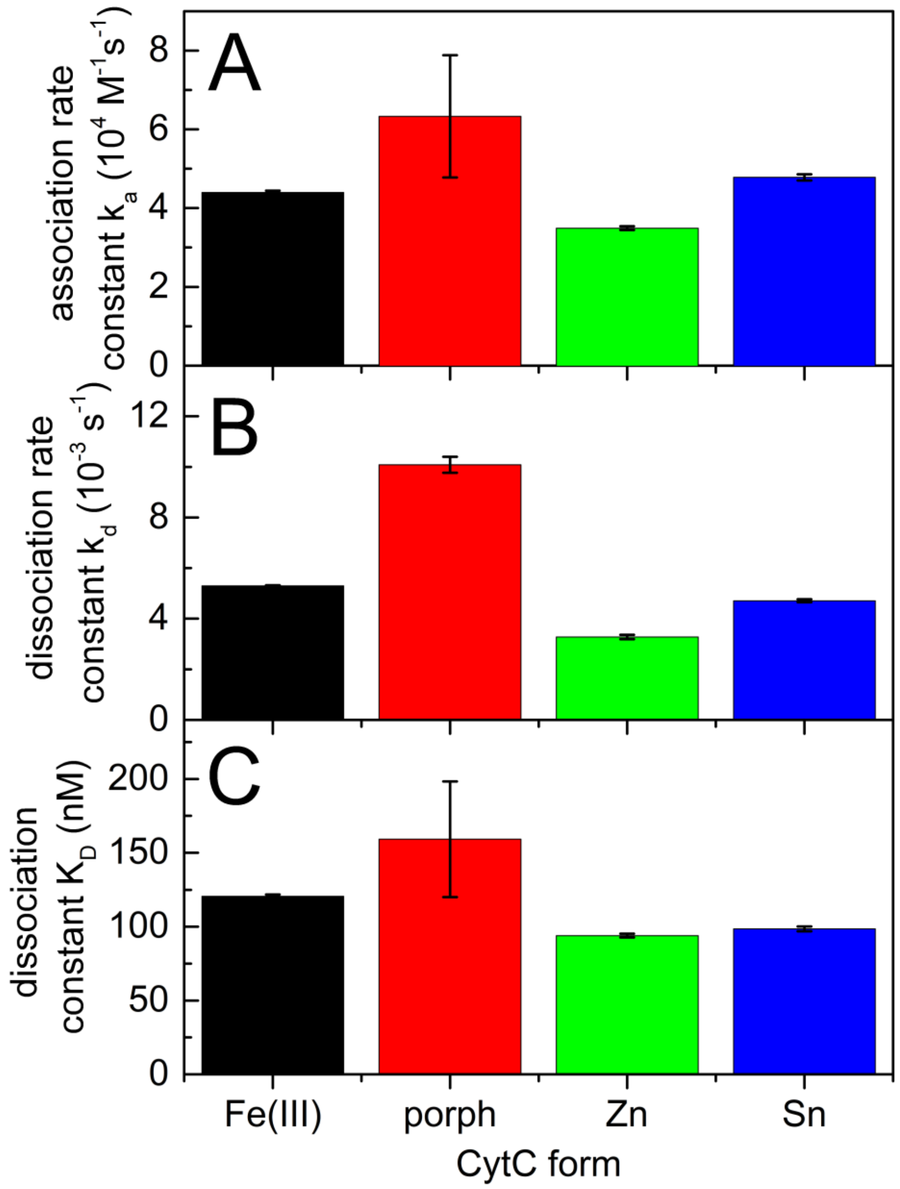

Fig. S17 Binding parameters of QD510-CytC pairs obtained by BLI experiments. QD510 was immobilized on Ni-NTA sensors (with His-tag-mCherry protein as linker) and binding of different CytC derivatives was measured. As control of unspecific binding sensors without QD510 immobilized were used. The binding constants were obtained from fitting of binding curves to the 1:1 binding model. The error bars represent standard errors of fitting of single measurement.

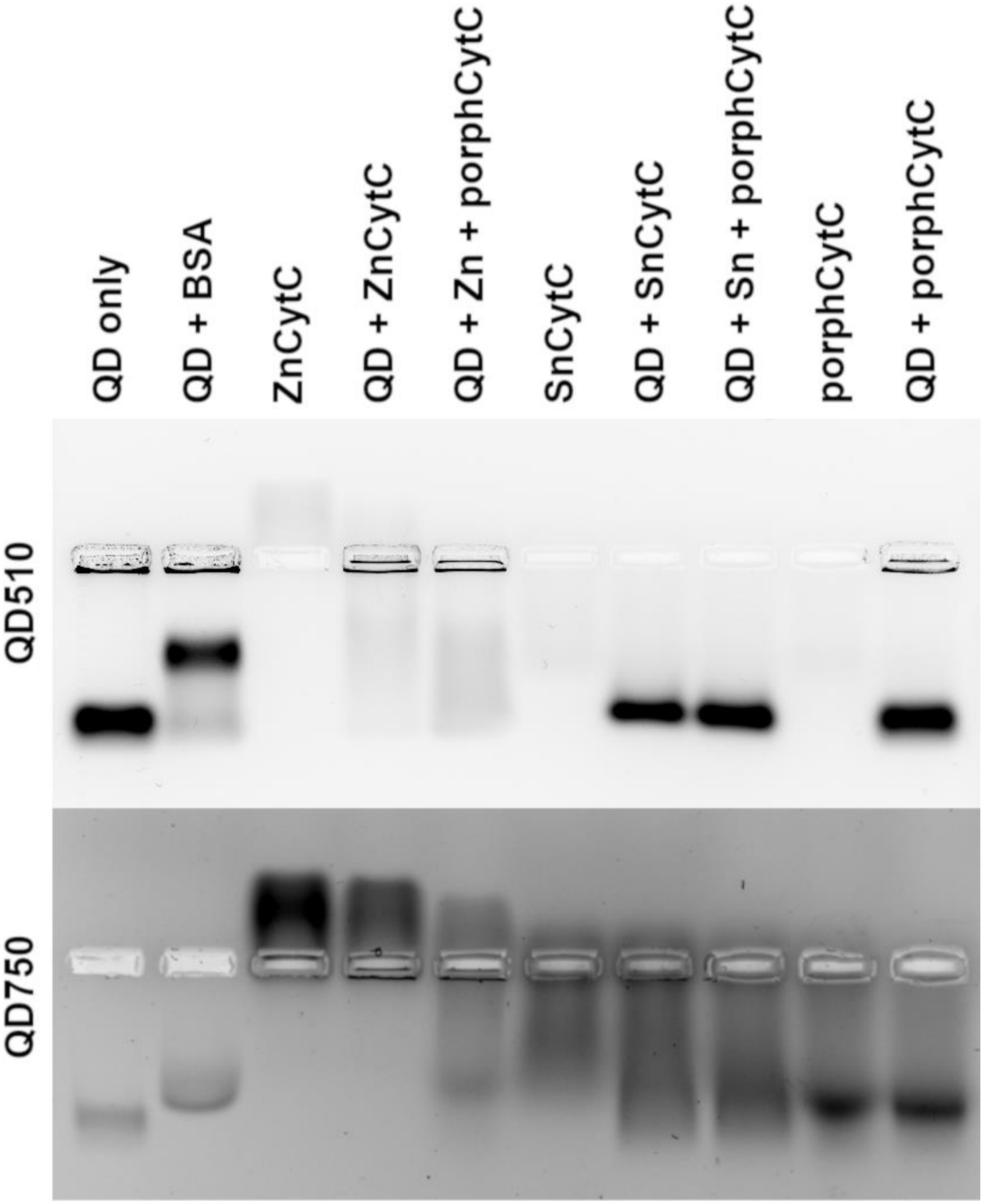

Fig. S18 Agarose gel electrophoresis of QD and CytC mixtures. Proteins were mixed in 1:2 or 100:1 protein:QD molar ratio with QD510 and QD750, respectively. Electrophoresis were performed in 1% agarose gel in 25 mM HEPES, pH 8.0 in constant 90 V voltage by approx. 40 min.

Supplementary references

1. Kapusta, P., Absolute diffusion coefficients: compilation of reference data for FCS calibration. . In *Application Note of PicoQuant GmbH, Berlin, Germany* 2010.
2. Burdzinski, G.; Bayda, M.; Hug, G.; Majchrzak, M.; Marciniec, B.; Marciniak, B., Time-resolved studies on the photoisomerization of a phenylene–silylene–vinylene type compound in its first singlet excited state. *J. Lumin.* **2011,** *131* (4), 577-580.
3. Yu, W. W.; Qu, L. H.; Guo, W. Z.; Peng, X. G., Experimental determination of the extinction coefficient of CdTe, CdSe, and CdS nanocrystals. *Chem. Mater.* **2003,** *15* (14), 2854-2860.
4. Vanderkooi, J. M.; Adar, F.; Erecińska, M., Metallocytochromes c: characterization of electronic absorption and emission spectra of Sn4+ and Zn2+ cytochromes c. *Eur. J. Biochem.* **1976,** *64* (2), 381-387.
5. Darżynkiewicz, Z. M.; Pędziwiatr, M.; Grzyb, J., Quantum dots use both LUMO and surface trap electrons in photoreduction process. *J. Lumin.* **2017,** *183*, 401-409.
6. Gopal, D.; Wilson, G. S.; Earl, R. A.; Cusanovich, M. A., Cytochrome c: ion binding and redox properties. Studies on ferri and ferro forms of horse, bovine, and tuna cytochrome c. *J. Biol. Chem.* **1988,** *263* (24), 11652-11656.
7. Grzyb, J.; Kalwarczyk, E.; Worch, R., Photoreduction of natural redox proteins by CdTe quantum dots is size-tunable and conjugation-independent. *RSC Advances* **2015,** *5* (76), 61973-61982.
8. Fluorophores and Their Amine-Reactive Derivatives. In *Molecular Probes™ Handbook. A Guide to Fluorescent Probes and Labeling Technologies*, 11th ed.; ThermoFisher: 2010; pp 11-97.
